# Pyridine Compounds with Antimicrobial and Antiviral Activities

**DOI:** 10.3390/ijms23105659

**Published:** 2022-05-18

**Authors:** Maria Marinescu, Claudia-Valentina Popa

**Affiliations:** 1 Department of Organic Chemistry, Biochemistry and Catalysis, Faculty of Chemistry, University of Bucharest, Soseaua Panduri, 030018 Bucharest, Romania; 2 Laboratory of Pharmaco-Toxicology, National Institute for Medical Military Research Development “Cantacuzino”, Splaiul Independenței No. 103, Sector 5, 050096 Bucharest, Romania

**Keywords:** pyridine, synthesis, heterocyclic compounds, antimicrobial, antiviral

## Abstract

In the context of the new life-threatening COVID-19 pandemic caused by the SARS-CoV-2 virus, finding new antiviral and antimicrobial compounds is a priority in current research. Pyridine is a privileged nucleus among heterocycles; its compounds have been noted for their therapeutic properties, such as antimicrobial, antiviral, antitumor, analgesic, anticonvulsant, anti-inflammatory, antioxidant, anti-Alzheimer’s, anti-ulcer or antidiabetic. It is known that a pyridine compound, which also contains a heterocycle, has improved therapeutic properties. The singular presence of the pyridine nucleus, or its one together with one or more heterocycles, as well as a simple hydrocarbon linker, or grafted with organic groups, gives the key molecule a certain geometry, which determines an interaction with a specific protein, and defines the antimicrobial and antiviral selectivity for the target molecule. Moreover, an important role of pyridine in medicinal chemistry is to improve water solubility due to its poor basicity. In this article, we aim to review the methods of synthesis of pyridine compounds, their antimicrobial and antiviral activities, the correlation of pharmaceutical properties with various groups present in molecules as well as the binding mode from Molecular Docking Studies.

## 1. Introduction

**Figure 1:**
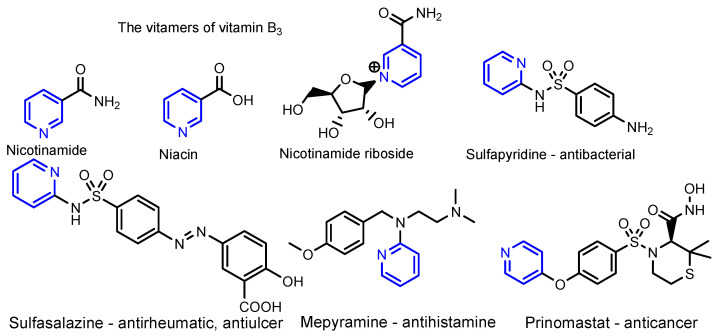
Drugs containing the pyridine nucleus.

Pyridine (from the Greek pyr = fire and idine—which is used for aromatic bases) contains a single heteroaromatic ring, which comes from the replacement of a CH group in the benzene ring with the nitrogen atom. Ramsay (1877) synthesizes pyridine for the first time, by the reaction of acetylene with hydrogen cyanide in a red-hot iron tube furnace, this being the first synthesized heterocycle [1]. Arthur Hantzsch later synthesized (1881) pyridine compounds by the synthesis that bears his name, through a multicomponent reaction, starting from a β-ketoester, an aldehyde and ammonia [2,3]. An important role of pyridine is that it is used as an organic solvent or as ligand for coordination complexes [4,5,6,7,8]. The pyridine nucleus is found in many natural products, such as vitamins, alkaloids and coenzymes, as well as in many drugs (Figure 1) and pesticides [9,10]. The recent literature mentions various series of compounds containing the pyridine nucleus, as a unique heterocycle, and with other heterocycles or fused with other heterocycles, which have remarkable antibacterial, antifungal and antiviral properties [1,9,10,11,12,13]. The presence of an additional heterocycle compared to the pyridine one in the bioactive molecule proved to be beneficial in the sense that it intensified the therapeutic properties of the antimicrobial and antiviral ones, and also widened their range [3,4,5,6,7,8]. Moreover, the presence of certain organic groups, such as amino, hydroxy, methoxy, sulfamide, hydrazide, etc., increase the biological activities of the compounds, as noted in the literature. Moreover, current research has been stimulated by the unexpected evolution of the SARS-CoV-2 pandemic (COVID-19), which required new antimicrobial and antiviral agents to treat patients with moderate or severe cases of pneumonia [13].

In the present paper, we aim to review antimicrobial and antiviral pyridine compounds, as well as their methods of synthesis. Their presentation will be based on the complexity of the molecules and their therapeutic properties.

## 2. Synthesis of Antimicrobial Compounds Containing Only Pyridine Ring

**Scheme 1:**
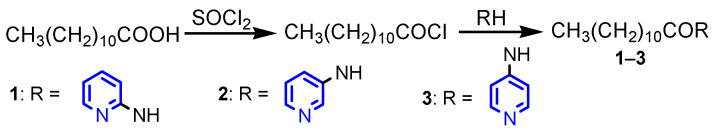
Synthesis of dodecanoic acid pyridines 1–3 [14].

Sarova et al. [14] synthesized three dodecanoic acid derivatives **1**−**3** with yields of 59–61**%**, starting from dodecanoic acid in two steps, chlorination with thionyl chloride and reaction with the corresponding aminopyridine (Scheme 1). All compounds possessed good antibacterial activity against *B. subtilis*, *S. aureus* and *E. coli* and antifungal activity against *A. niger* and *C. albicans*.

**Scheme 2:**
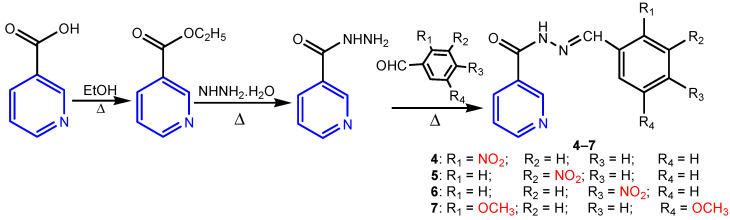
Synthesis of nicotinic acid benzylidene hydrazide derivatives **4**–**7** [15].

Narang et al. [15] synthesized a series of 18 nicotinic acid benzylidene hydrazide derivatives in three steps with total yields of 60–80% (Scheme 2). The antimicrobial screening results indicated that compounds with nitro (**4**, **5** and **6**) and dimethoxy (**7**) substituents were the most active ones against tested strains (*S. aureus*, *B subtilis*, *E coli*, *C. albicans*, and *A. niger*), some of them having an antimicrobial activity comparable to the standard drugs fluconazole and norfloxacin.

**Scheme 3:**
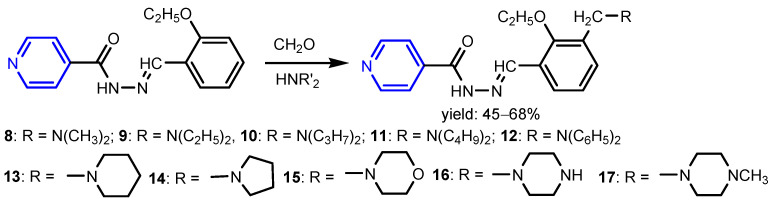
Synthesis of Mannich bases **8**–**17** [16].

**Scheme 4:**
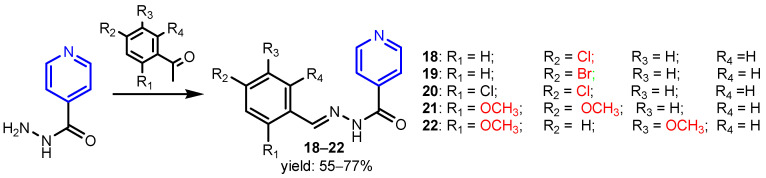
Synthesis of isonicotinic acid-1-(substituted phenyl)-ethylidene hydrazides **18**–**22** [17].

Malhotra et al. [16] synthesized ten substituted Mannich bases **8**–**17** using a classical Mannich reaction starting from 2-ethoxybenzylidene isonicotinohydrazide, formaldehyde and a secondary amine (Scheme 3). The highest antibacterial activity against Gram-positive species *B. subtilis*, *S. aureus* and Gram-negative species *P. aeruginosa, E. coli* were shown by compounds **12**, **15**, **16**, and **17** (MIC = 6.25–12.5 μg mL^−1^) with Amoxicillin as standard. Furthermore, these compounds exhibited a high antifungal activity against *C. albicans* and *C. gabrata* species (MIC = 12.5 μg mL^−1^). Judge et al. [17] synthesized isonicotinic acid-1-(substituted phenyl)-ethylidene hydrazides **18**–**22** by refluxing isonicotinoyl hydrazide with various substituted acetophenones (Scheme 4). Compounds with Br, OCH_3_, and Cl groups were highly active antimicrobial agents, all of them exhibiting activities better than the standard compounds norfloxacin and fluconazole.

**Scheme 5:**
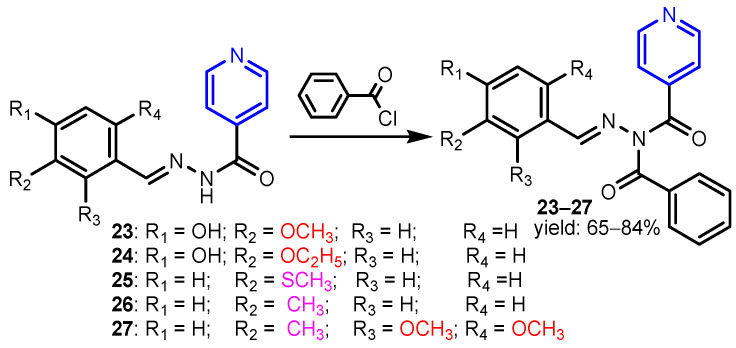
Synthesis of isonicotinic acid hydrazide benzoyl derivatives **23**–**27** [18].

**Figure 2:**
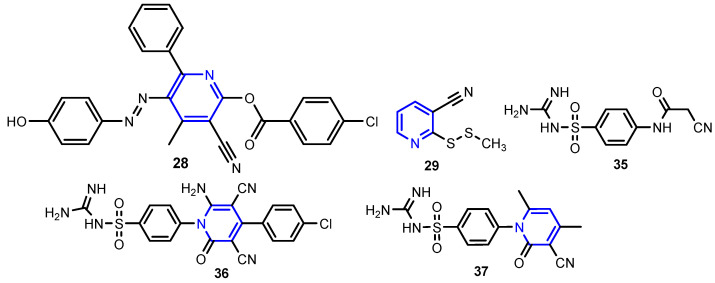
Structure of pyridine compounds **28**, **29**, **36**, **37,** and intermediate **35** [19,20,22].

**Scheme 6:**
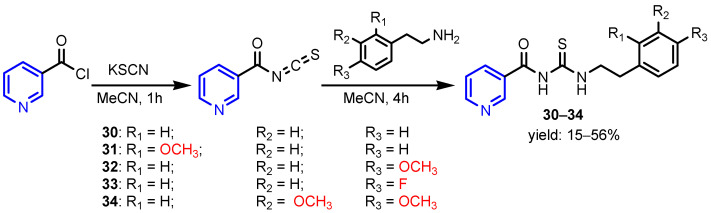
Synthesis of pyridine compounds **30**–**34** [21].

In a similar study, Judge et al. [18] synthesized a series of isonicotinic acid hydrazide derivatives by benzoilation of (*E*)-N’-benzylideneisonicotinohydrazide compounds (Scheme 5) and found that derivatives **23**–**27** were the most active antimicrobial agents against the tested strains *S. aureus*, *B. subtilis*, *E. coli*, *C. albicans*, and *A. niger*, with MIC between 2.18–3.08 μM mL^−1^. Eldeab [19] synthesized substituted pyridyl-4-chloro benzoates by microwave-assisted condensation of 2-oxo-1,2-dihydropyridine-3- carbonitriles with 4-chlorobenzoyl chloride under solvent-free conditions or by conventional heating in methylene chloride in the presence of triethylamine. The activity of 3-cyano-5-[(4-hydroxyphenyl)diazenyl]-4-methyl-6-phenylpyridin-2-yl-4-chloro benzoate **28** against *B. subtilis* and *S. aureus* was comparable to that of Amikacin, which is used as the standard antibiotic (Figure 2). Karunanidhi et al. [20] isolated 2-(methyldithio)pyridine-3-carbonitrile **29** from Persian Shallot (*Allium stipitatum* Regel.) and screened for its antimicrobial activity. The minimum inhibitory concentrations (MICs) were found to be in the range of 0.5 to 64 μg mL^−1^ for the tested bacterial strains (*A. baumannii*, *A. iwoffii*, *Enterobacter* sp., *E. coli*, *S. aureus*, *P. aeruginosa*, *S. typhi*, *S. dysenteriae* and *S. maltophilia*) and of 0.25 to 2 μg mL^−1^ for *Candida* species (Figure 2). Özgeriş [21] synthesized nicotinoyl compounds **30**–**34** by reaction of nicotinoyl chloride with potassium thiocyanate or ammonium thiocyanate to get nicotinoyl isothiocyanate, which refluxed with substituted phenethylamines in acetone to yield the nicotinoyl thioureas (Scheme 6). All compounds showed excellent antibacterial activity against the tested bacterial strains *S. aureus*, *E. faecalis*, *E. coli*, *A. baumannii*, and *P. aeruginosa* with MIC concentrations of 31.25 to 62.5 μg mL^−1^. Ragab et al. [22] reported the synthesis of pyridine compounds **36** (by ternary condensation of cyanoacetamide **35**, aromatic aldehyde and malononitrile in an equimolar molar ratio 1:1:1 in basic ethanol) and **37** (by cyclo-condensation of cyanoacetamide **35** with acetylacetone in DMF) with very good antimicrobial activity against all tested strains *B. subtilis*, *S. aureus*, *E. faecalis*, *E. coli*, *P. aeruginosa*, *S. typhi*, *C. albicans,* and *F. oxysporum* (MIC = 18–31 μM) (Table 1).

**Scheme 7:**
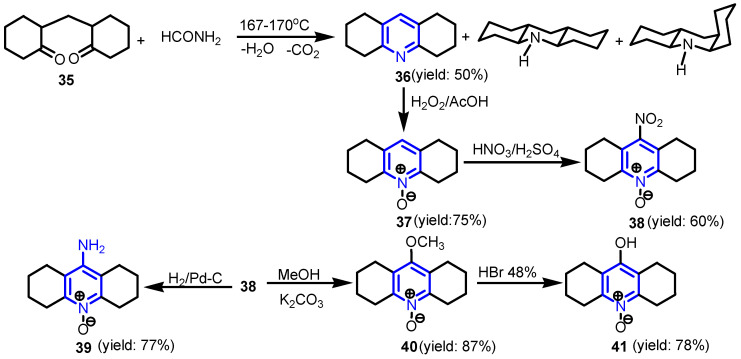
Synthesis of 1,2,3,4,5,6,7,8-octahydroacridine-10-oxides **38**–**41** [23,24,25,26,27,28].

Marinescu et al. [23,24,25,26,27,28] synthesized a series of tetrasubstituted pyridine-N-oxides (octahydroacridines) **38–41**, starting from the reaction of 2,2′-methylenedicyclohexanone with formamide at 167–170 °C and the separation of 1,2,3,4,5,6,7,8-octahydroacridine **36** from the reaction mixture (Scheme 7). Oxidation of compound **36** with hydrogen peroxide and acetic acid gave the key intermediate **37**, *sym*-octahydroacridine-10-oxide, which, through reactions of nitration, reduction, alkylation, and hydrolysis, gave the compounds **38**, **39**, **40,** and **41**. It was found that antimicrobial activity against the tested pathogens *S. epidermidis* 1736, *S. aureus* ATCC 25923, *E. faecalis* ATCC 29212, *B. subtilis* 6688, *P. stuartii* 1116, *P. aeruginosa* 846, *K. pneumoniae* ESBL+, *S. marcenscens* 0804, *C. freundii* 1748, *E. coli* 1576, ESBL+, *Salmonella* sp. 9246, *C. albicans* ATCC 10231 increased in the order **40** < **39** < **38** < **41**, simultaneously with the decrease of the hydration energy from 0.59, to 1.72, to 3.14 and respectively to 3.76 kcalMol^−1^.

## 3. Synthesis of Antimicrobial Pyridine Salts

**Scheme 8:**
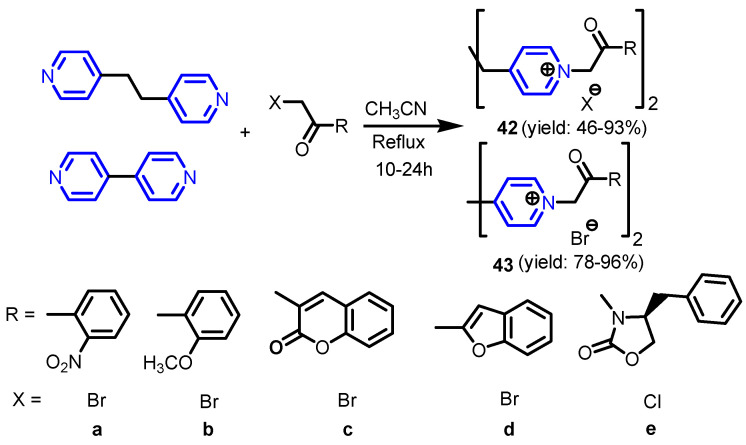
Synthesis of bis-pyridinium quaternary ammonium salts [29].

**Scheme 9:**
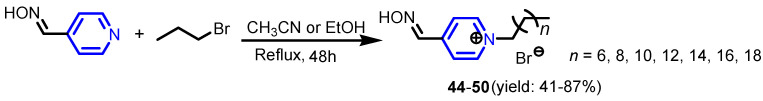
Synthesis of pyridine-4-aldoxime-based quaternary ammonium salts [30].

Furdui et al. [29] reported efficient synthesis of symmetrical diquaternary salts by alkylation of 4-[2-(pyridin-4-yl)ethyl]pyridine or 4,4′-bipyridine, with various bromo- or chloro-acetophenone analogues (Scheme 8) and investigated their antimicrobial activity against nine different microorganisms: *B. subtilis*, *B. cereus*, *S. lutea*, *R. glutinis*, *C. utilis*, *S. cerevisiae*, *A. niger*, *G. candidum* and *P. roqueforti*. Compounds **42a**–**42d**, **43a** and **43d** show efficient inhibitory properties at least against one bacterial strain. Marek et al. [30] synthesized a set of pyridine-4-aldoxime-based quaternary ammonium salts with differing lengths of alkyl side chains **44**–**50** (Scheme 9). The in vitro antibacterial activity of all compounds was tested on a panel of eight bacterial strains (*S. aureus* CCM 4516/08, *S. aureus* MRSA H 5996/08, *S. epidermidis* HK6966/08, *Enterococcus* sp. HK14365/08, *E. coli* CCM 4517, *K. pneumoniae* D 11750/08, *K. pneumoniae* J 14368/08, and *P. aeruginosa* CCM 1961), four yeasts (*C. albicans* ATCC 44859, *C. krusei* E28, *C. tropicalis* 156, *C. glabrata* 20/I) and four filamentous fungi (*Trichosporon asahii* 1188, *Aspergillus fumigatus* 231, *Absidia corymbifera* 272, *Trichophyton mentagrophytes* 445). The compounds with an alkyl chain of C12–C16 possessed the best antimicrobial activity of all.

**Scheme 10:**
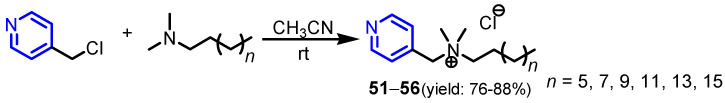
Synthesis of pyridine salts **51**–**56** [31].

Brycki et al. [31] performed a Menshutkin reaction between 4-chloromethylpyridine and the N,N-dimethylalkylamines containing 8, 10, 12, 14, 16, 18 carbon atoms in an alkyl chain, respectively in acetonitrile to obtain compounds **51**–**56** in good yields at room temperature (Scheme 10). The minimal inhibitory concentration (MIC) values of pyridine salts **51**–**56** against the fungi *A. niger*, *C. albicans*, *P. chrysogenum* range from 0.1 to 12 mM and against bacteria *S. aureus*, *B. subtilis*, *E. coli*, *P. aeruginosa* range from 0.02 to 6 mM, as can be seen in Table 2.

**Figure 3:**
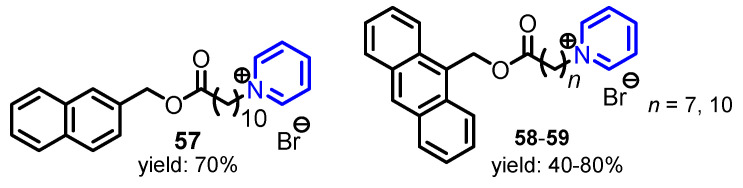
Structure of pyridine salts **57**–**59** [32].

Wang et al. [32] showed that pyridinium bromide salts **57**–**59** (Figure 3) exhibited excellent antibacterial activity against three Gram-negative bacteria *Xanthomonas oryzae*, *Ralstonia solanacearum*, and *Xanthomonas axonopodis*, because of the appropriate balance between hydrophobicity and hydrophilicity in these molecules.

**Scheme 11:**
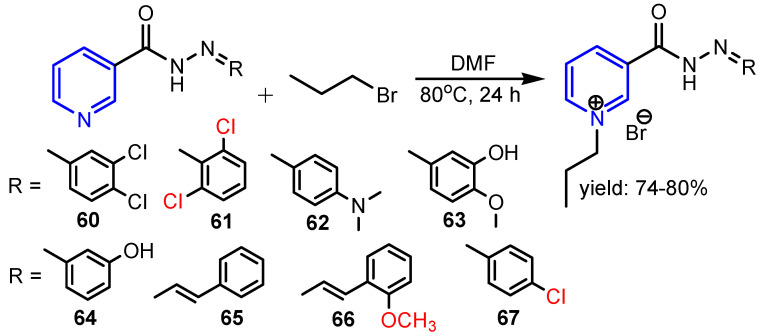
Synthesis of pyridine salts **60**–**67** [33].

Ali et al. [33] reported synthesis of eight N-alkylated pyridine-based organic salts **60**–**67** (Scheme 11) and their antibacterial and antibiofilm activities. The best antibacterial activity was shown by compound **66** at the concentration of 100 μg mL^−1^ with MIC values of 56 ± 0.5% against *S. aureus* and 55 ± 0.5% against *E. coli*; followed by salt **65** (MIC = 55 ± 0.5% against *E. coli*) and **61** (MIC = 55 ± 0.5% against *E. coli*) at the same concentration. In the antibiofilm activity, salts **65**, **60** and **67** inhibited 58 ± 0.4% *S. aureus* at a concentration of 75 μg mL^−1^, while **61** inhibited 55 ± 0.5% *E. coli* at concentration of 100 μg mL^−1^.

**Figure 4:**
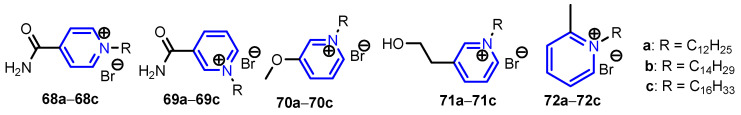
Structure of pyridine salts **68**–**72** [34].

**Scheme 12:**
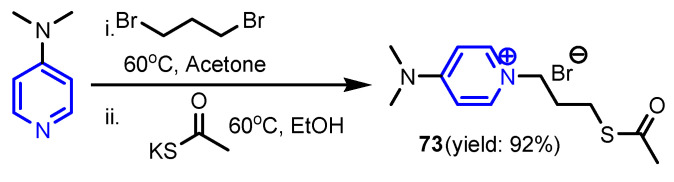
Synthesis of 4-(dimethylamino)pyridinepropylthioacetate **73** [35].

Soukup et al. [34] synthesized five series of pyridinium salts **68a**–**68c**, **69a**–**69c**, **70a**–**70c, 71a**–**71c** and **72a**–**72c** via an S_N_2 type Menshutkin reaction (Figure 4) with yields of 81–98%. Compound **72c** showed a rather versatile efficacy against the whole spectrum of microbes, three Gram-positive (*S. aureus* C1947, methicillin-resistant *S. aureus* MRSA C1926, vancomycin-resistant Enterococcus S2484), six Gram-negative strains (*E. coli* A1235, *K. pneumoniae* C1950, *K. pneumoniae* C1934, *A. baumannii* J3474, *Stenotrophomonas maltophilia* J3552, *Yersinia bercovieri* CNCTC 6230), and eight fungal strains, four yeasts (*Candida parapsilosis sensu stricto* EXF-8411, *Rhodotorula mucilaginosa* EXF-8417, *Exophiala dermatitidis* EXF-8470, *Aureobasidium melanogenum* EXF-8432) and four filamentous fungi (*Bisifusarium dimerum* EXF-8427, *Penicillium chrysogenum* EXF-1818, *Aspergillus versicolor* EXF-8692, and *Aspergillus niger* EXF-10185). Anwar et al. [35] reported synthesis of 4-(dimethylamino)pyridinepropylthioacetate **73** (Scheme 12) and effects of **73** coated with gold nanoparticles **73**-AuNPs against *E. coli* (ATCC 8739). The MIC of the Pefloxacin + **73**-AuNPs mixture was found to be 25 μg mL^−1^ as compared to Pefloxacin alone (50 μg mL^−1^), which clearly indicates that **73**-AuNPs increased the potency of Pefloxacin.

## 4. Synthesis of Antimicrobial Pyridine Compounds Containing a Five-Membered Ring with One or Two Heteroatoms

**Figure 5:**
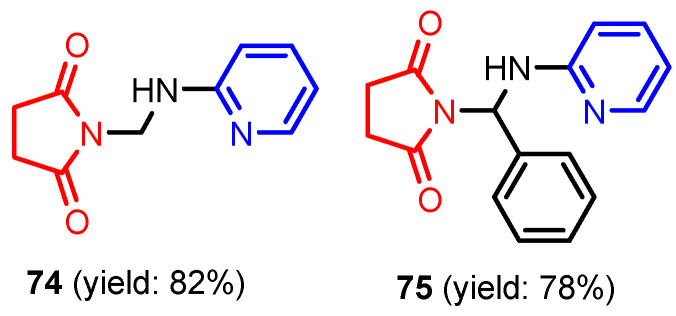
Structure of Mannich pyrol-pyridine bases **74** and **75** [36].

Tamilvendan et al. [36] synthesized two Mannich pyrol-pyridine bases 1-((pyridin-2-ylamino)methyl)pyrrolidine-2,5-dione **74** and 1-(phenyl(pyridin-2-yl amino)methyl) pyrrolidine-2,5-dione **75** using a classical Mannich reaction between succinimide, aniline, and formaldehyde or benzaldehyde (Figure 5) in good yields (78–80%). Both compounds showed moderate antimicrobial activity against the antibacterial panel (*Escherichia coli*, *Salmonella typhi*, and *Bacillus subtilis*) and antifungal agents (*Aspergillus oryzae* and *Aspergillus fumigates*), using Penicillin, Streptomycin, and Amphotericin B as standards.

**Scheme 13:**
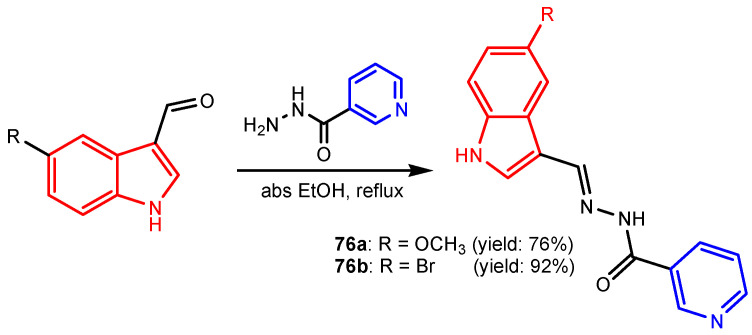
Synthesis of pyridine-indole compounds **76a** and **76b** [37].

**Scheme 14:**
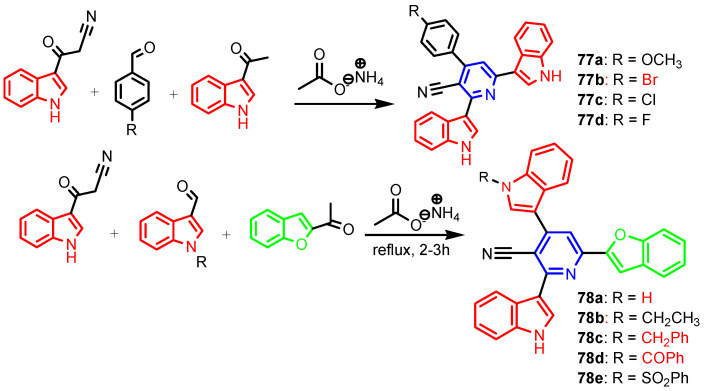
Synthesis of bis(indolyl)pyridines **77a–77d** and **78a–78e** [38].

**Figure 6:**
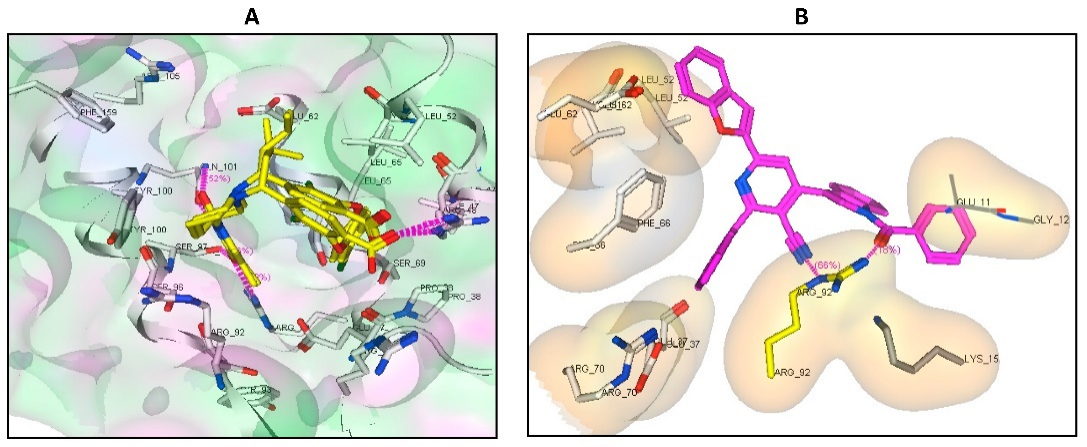
(**A**) The binding mode of the re-docked ligand within the binding pocket of TMPK (ID: 4QGG). The re-docked ligand shows superimposed on the same position as the native ligand (yellow, stick) with the same orientation. The two ligands show the same H-bonds profile with Gln101, Arg48, Arg70, and Ser97 (white, stick). (**B**) The binding mode of compound **78d** (pink, stick) within the binding pocket of TMPK (ID: 4QGG). Compound **78d** shows π-cation interactions and two H-bound acceptors with the amine acid residue Arg92 (yellow, stick) [38].

**Scheme 15:**
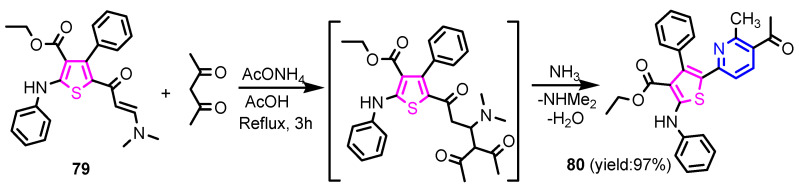
Synthesis of thiophene-pyridine compound **80** [39].

**Scheme 16:**
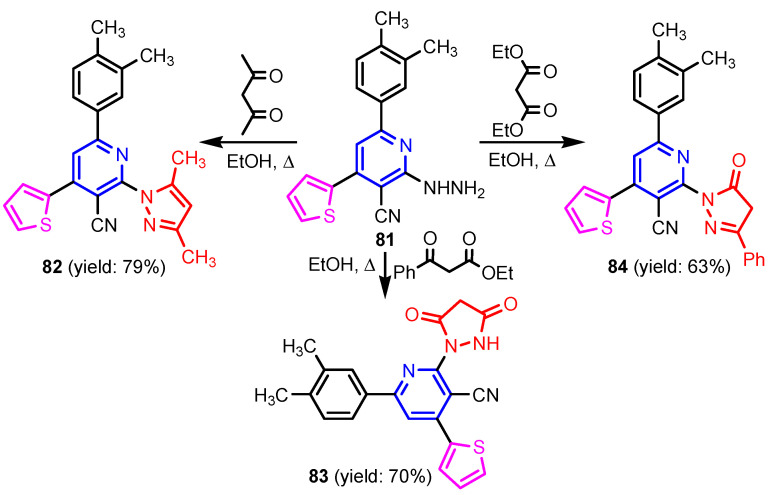
Synthesis of thiophene-pyrazole-pyridine compounds **82**–**84** [40].

**Figure 7:**
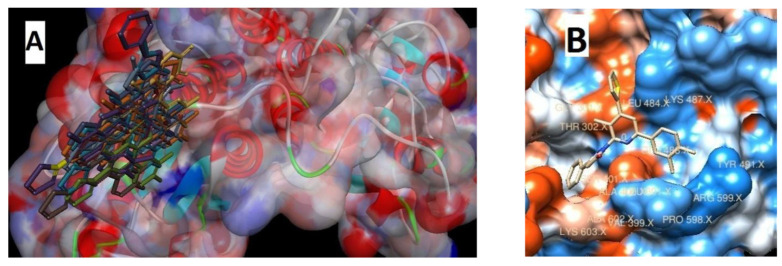
Docking into the active site of glucosamine-6-phosphate (GlcN-6-P) synthase (PDB ID: 2VF5). (**A**) Docked poses of compounds **82**–**84** on the GlcN-6-P; (**B**) the interaction the compound **84** and GlcN-6-P [40].

**Scheme 17:**
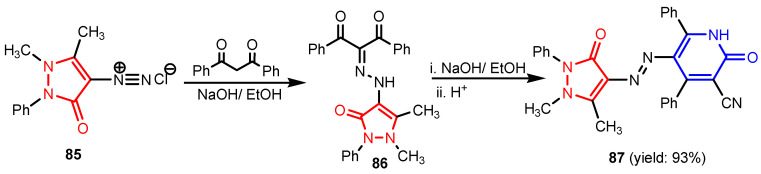
Synthesis of phenyldiazenyl pyridone **87** [41].

**Scheme 18:**
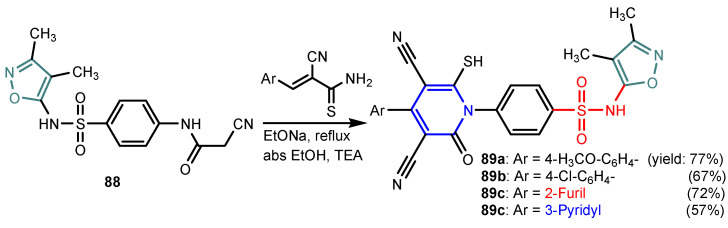
Synthesis of isoxazol-pyridonethiols **89a–89d** [42].

**Scheme 19:**
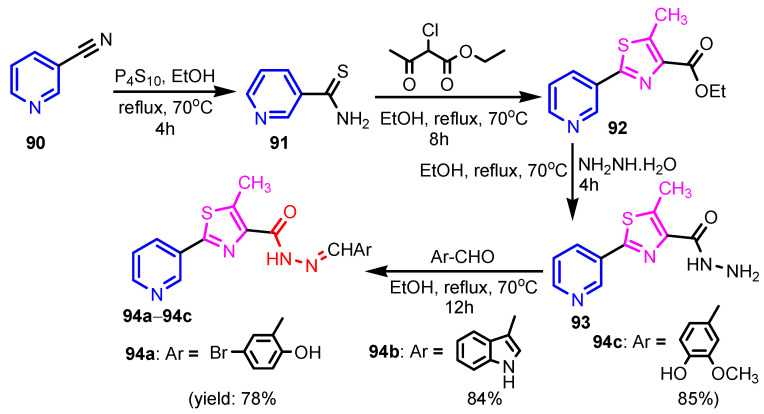
Synthesis of thiazole-pyridine hybrids **94a**–**94c** [43].

**Figure 8:**
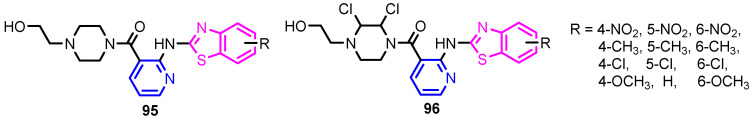
Structures of pyridine-benzothiazole hybrids **95** and **96** [44].

**Scheme 20:**
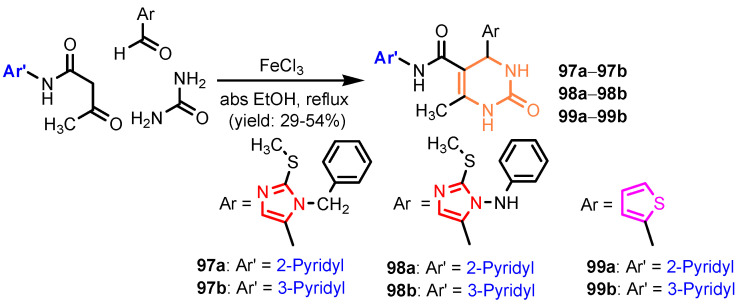
Synthesis of pyrimidines **97**, **98** and **99** [45].

**Figure 9:**
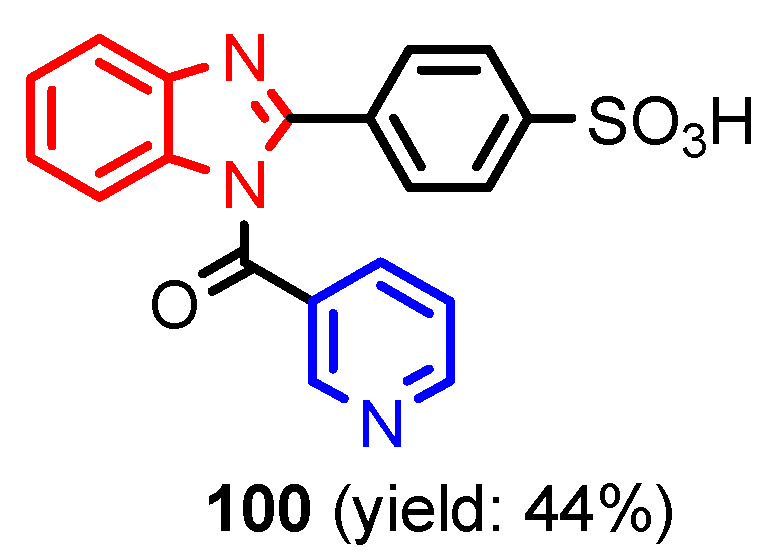
Structure of benzimidazole-pyridine **100** [46].

**Scheme 21:**
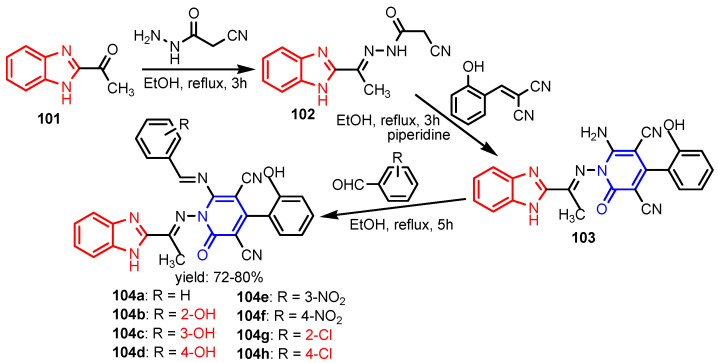
Synthesis of benzimidazole-2-pyridone hybrids **104a**–**104h** [47].

**Scheme 22:**
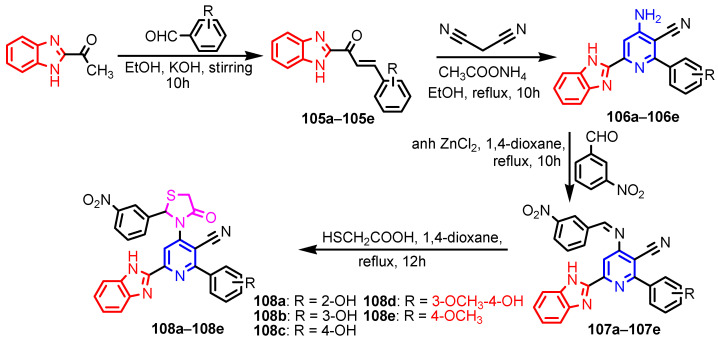
Synthesis of benzimidazole-cyanopyridine—thiazolidinones **108a–108e** [48].

**Figure 10:**
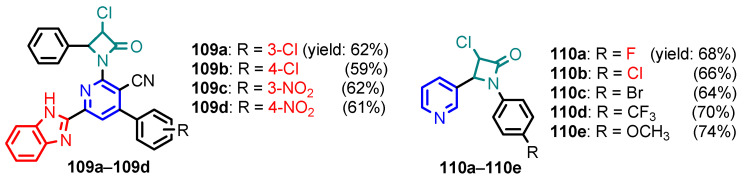
Structure of benzimidazole-pyridine **109a**–**109d** and **110a**–**110e** [49,50].

**Scheme 23:**
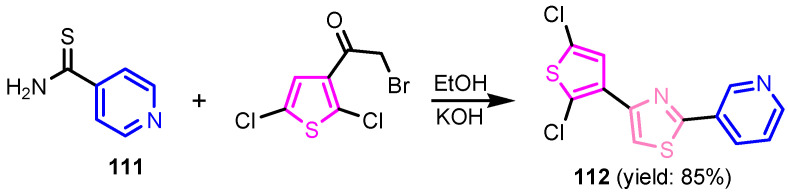
Synthesis of 4-(2,5-dichlorothiophen-3-yl)-2-(pyridin-3-yl)thiazole **112** [51].

**Scheme 24:**
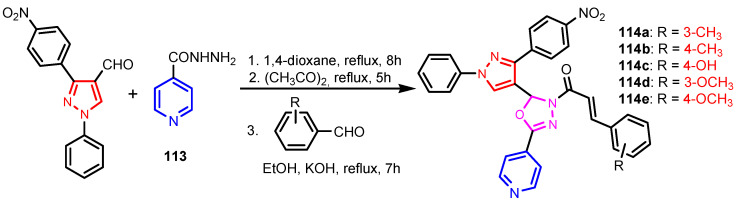
Synthesis of pyrazole bearing pyridyl oxadiazole analogues **114a**–**114e** [52].

**Figure 11:**
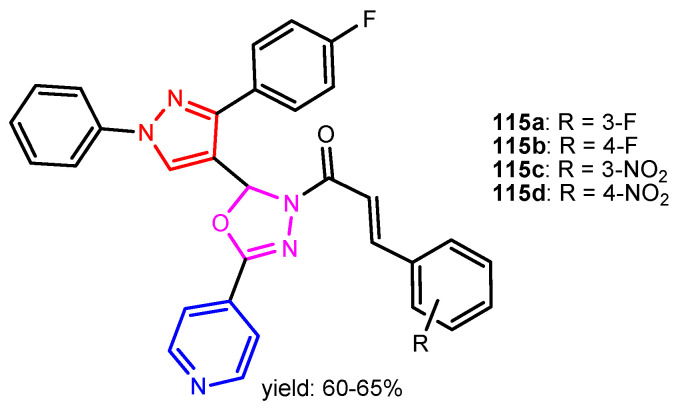
Pyridyl-pyrazole-1,3,4-oxadiazoles **115a**–**115d** [53].

Angelova et al. [37] reported the synthesis of two pyridine-indole compounds **76a** and **76b** by condensation of nicotinohydrazide with 5-methoxy-1*H*-indole-3-carbaldehyde or 5-bromo-1*H*-indole-3-carbaldehyde in an ethanol solution, and evaluated their in vitro antibacterial activity against *M. tuberculosis* H37Rv (MIC = 1.6989 μM and 2.9139 μM respectively) using isoniazide and ethambutol as reference drugs (Scheme 13). Abo-Salem et al. [38] synthesized a new class of bis(indolyl)pyridines **77** analogues of the marine alkaloid nortopsentin in good yields (75–96%), using a one-pot four-component condensation of 3-cyanocarbomethylindole, various aldehydes, 3-acetylindole, and ammonium acetate in glacial acetic acid. Moreover, 2,6-bis(1*H*-indol-3-yl)-4-(benzofuran) pyridine-5-carbonitriles **78** were synthesized via a one-pot four-component condensation of 3-cyanocarbomethyl indole, 2-acetylbenzofuran, N-substituted-indole-3-aldehydes and ammonium acetate (Scheme 14). Four compounds—**77b**, **78a**, **78c**, and **78d**—demonstrated good antimicrobial activity against *S. aureus*, *E. coli*., and *C. albicans*, as can be seen in Table 3. All antimicrobial active compounds studied (**77b**, **78a**, **78c**, and **78d**) revealed favorable binding interactions and binding energy with the binding site of thymidylate kinase (ID: 4QGG) with values ranging from −15.52 to −25.36 kcal mol^−1^, which is comparable to the native ligand of −24.34 kcal mol^−1^. Figure 6 revealed the binding mode of the re-docked **78d** within the binding pocket of TMPK (Thymidylate kinase, ID: 4QGG). The structures of Thymidylate kinase (TMPK), DNA gyrase subunit B, and DNA topoisomerase IV subunit B were retrieved from the protein data bank at http://www.rscb.org./pdb (accessed on 1 May 2022) using 4QGG, 6F86, 4HZ5 codes, respectively, and a Gaussian Contact surface around the binding sites were drawn using MOE 2008.10 (Molecular Operating Environment, http://www.chemcomp.com (accessed on 1 May 2022). Mabkhot et al. [39] reported synthesis of thiophene-pyridine compound **80** by reaction of the enaminone **79** with pentane-2,4-dione in glacial acetic acid in the presence of ammonium acetate (Scheme 15). The presence of the pyridine nucleus has been shown to be necessary for better antifungal activity than Amphotericin B (standard) against Aspergillus fumigates and Syncephalastrum racemosum. Moreover, compound **80** revealed a moderate activity towards Geotricum candidum and Candida albicans. Flefel et al. [40] reported the synthesis of thiophene-pyrazole-pyridine hybrids **82**–**84**, by reaction between thiophene-pyridine **81** and carbonilic compounds (Scheme 16). All synthesized compounds possessed good antimicrobial activity against almost all tested strains, *Staphylococcus aureus*, *Bacillus subtilis*, *Escherichia coli*, *Salmonella typhimurium*, *Aspergillus flavus*, and *Candida albicans*. Figure 7 shows the docked images of the drug candidates **82**–**84** on the target protein. The results of in silico studies revealed a moderate-to-good pattern of affinity and activity of these compounds on the target protein GlcN-6-P synthase, which is supported by in vitro antimicrobial screenings [40]. In this study, a Lamarckian genetic algorithm contained in AutoDock (version 4.0) was employed for in silico molecular docking studies. Alnassar et al. [41] synthesized phenyldiazenyl pyridone **87** starting from diazopyrazolone **85** in two steps: condensation with 1,3-diphenylpropane-1,3-dione in alkaline ethanole to give intermediate **86**, and cyclisation of **86** in acidic medium (Scheme 17). The antibacterial activity revealed that compound **87** was active against two tested strains, *Bacillus subtilis* ATCC 6633 and *Escherichia coli* ATCC 25922. Nasr et al.; [42] synthesized izoxazole-pyridone derivatives by refluxing cyanothioacetamide **88** solution with the corresponding arylidene derivatives in absolute ethanol (Scheme 18). All compounds have good antimicrobial activity against all tested strains, *S. pneumoniae*, *B. subtilis*, *S. epidermidis*, *E. coli*, *P. vulgaris*, and *K. pneumonia*. The pyridonethiols **89b** and **89c** showed double potency of Ampicillin in inhibiting the growth of *B. subtilis* (MIC = 0.12 μg mL^−1^ vs 0.24 μg mL^−1^), while compound **89d** was equipotent to Ampicillin against the same bacteria. Kamat et al. [43] reported a synthesis of thiazole-pyridine hybrids **94a**–**94c** in four steps, starting from nicotinonitrile (Scheme 19). The in vitro antimicrobial assay showed that compounds **94** are effective against all tested strains *S. aureus*, *B. subtilis*, *E. coli*, *P*. *aeruginosa, A. flavus, T. atroviridae, P. citranum*, and *C. albicans* with safe pharmacokinetic properties. Patel et al. [44] synthesized two series of pyridine-benzothiazole hybrids **95** and **96**, by reaction between 2-[N-(substitutedbenzo thiazolyl)amino]pyridine-3- carbonyl chlorides and 2-(piperazin-1-yl)ethanol or 2,3-dichloropiperazine, respectively with yields of 55–74% (Figure 8). It was found that all compounds have a poor to fair activity against all tested strains, *P. aeruginosa*, *E. coli*, *S. aureus*, *B. subtilis*, and *C. albicans*. Sedaghati et al. [45] synthesized novel pyrimidines **97**, **98**, and **99** by a classic Biginelli reaction, using FeCl_3_ as a catalyst (Scheme 20). Most of the compounds had better antifungal than antibacterial activity against the tested microorganisms (*E. coli*, *S. enteritidis*, *P. aeruginosa*, *S. aureus*, *L. monocytogenes*, *B. subtilis*, *A. niger*, *C. albicans*). Moderate antifungal agents, **97a**, **98a**, and **98b** were determined among the studied compounds. Yadav et al. [46] reported benzimidazole-pyridine **100** synthesis with weak antimicrobial activity against tested strains, *S. aureus*, *B. subtilis*, *E. coli*, *C. albicans*, and *A. niger*, using Ciprofloxacin and Fluconazole as control drugs (Figure 9). Desai et al. [47] reported synthesis of benzimidazole-2-pyridone hybrids **104** using the reactions shown in Scheme 21. Condensation of 1-(1H-benzo[d]imidazol-2-yl)ethanone **101** with 2-hydrazinylacetyl cyanide in alcohol gave hydrazinylacetylcyanide **102**, which is cyclized in the second step with 2-(2-hydroxy-benzylidene)malononitrile in the presence of a catalytic amount of piperidine to furnish dihydropyridine-3,5-dicarbonitrile **103**. Finally, **103** is condensed with different aryl aldehydes to form the final compounds **104a**–**104h**. Compounds bearing chloro and hydroxy groups exhibit very good to excellent antimicrobial activity against all tested strains, *S. aureus*, *S. pyogenes*, *E. coli*, *P. aeruginosa*, *C. albicans*, *A. niger*, and *A. clavatus*. A new series of benzimidazole derivatives bearing cyanopyridine and 4-thiazolidinone motifs were synthesized by Desai et al. [48] in four steps, starting by condensation of 1-(1*H*-benzo[d]imidazol-2-yl)ethanone with aromatic aldehydes to get compounds **105a**–**105e**, which were treated with ammonium acetate and malononitrile to achieve derivatives **106a**–**106e**, produced via a Knoevenagel condensation reaction (Scheme 22). Compounds **106** underwent simple condensation reactions with 3-nitrobenzaldehyde to provide Schiff bases **107a**–**107e**, which in the last step generated final analogs **108a**–**108e** using cyclization with thioglycolic acid. As can be seen in Table 4, the antimicrobial activities of the compounds **108a**–**108e** were good to excellent, compound **108d** having better antimicrobial activities than the standards used—Chloramphenicol and Ketoconazole—in most cases, followed by compound **108e**, which proves that the presence of the methoxy group and the hydroxy group simultaneously, or, in the case of the methoxy group, leads to a better biological activity. Continuing the research, Desai et al. [49] reported the synthesis of 2-azetidinones **109** from the reaction of compounds **107** with chloroacetyl chloride in 1,4-dioxane. Compounds **109a**–**109d** (Figure 10) showed excellent antimicrobial activity compared to the standards used—Chloramphenicol and Ketoconazole. Furthermore, Rani and Reddy [50] reported the synthesis and antimicrobial activity of pyridine containing substituted phenyl azetidine-2-ones **110**. Compounds 3-chloro-1-(4-fluorophenyl)/(4-chlorophenyl)-4- (pyridine-3-yl) azetidine-2-one (**110a** and **110b**) were found to be more potent against all tested strains, *S. aureus*, *B. subtilis*, *E. coli*, *P. aeruginosa*, *A. niger*, and *P. rubrum*. Sarojini et al. [51] reported synthesis of 4-(2,5-dichlorothiophen-3-yl)-2-(pyridin-3-yl) thiazole **112** by reaction of pyridine-4-carbothioamide **111** with 2-bromo-1-(2,5-dichlorothiophen -3-yl)ethanone in ethanolic KOH (Scheme 23). Compound **112** had a weak antimicrobial activity against the tested strains, *S. aureus* and *P. aeruginosa* (MIC = 100 μg mL^−1^), compared to the standard medicine Ampicillin (MIC< 6.5 mL^−1^). Desai et al. [52] reported the synthesis of pyrazole bearing pyridyl oxadiazole analogues **114a**–**114e** starting from 3-(4-nitrophenyl)-1- phenyl-1*H*-pyrazole-4-carbaldehyde, in three steps: 1. condensation, with isonicotino hydrazide **113** with formation of hydrazide; 2. cyclization reaction with acetic anhydride under reflux condition, with the formation of the 1,3,4-oxadiazole; 3. Claisen-Schmidt condensation of 1,3,4-oxadiazole intermediate with aromatic aldehydes in ethanolic potassium hydroxide (Scheme 24). All compounds showed considerable inhibition against nearly all of the tested bacteria and fungi as compared to the reference drugs, Chloramphenicol and Griseofulvin, as can be observed in Table 5. Following the same synthetic steps, but starting from 3-(4-fluorophenyl)-1-phenyl-1*H*-pyrazole-4- carbaldehyde, Desai et al. [53] synthesized fluorinated pyrazole encompassing pyridyl 1,3,4-oxadiazole motifs **115a**–**115d** (Figure 11). All compounds showed considerable inhibition against nearly all of the tested bacteria, and were proved to be potential antifungal agents, superior to the reference drug (Griseofulvin) at low level of cytotoxic concentrations.

## 5. Synthesis of Antimicrobial Pyridine Compounds Containing a Five-Membered Ring with Three Heteroatoms

**Scheme 25:**
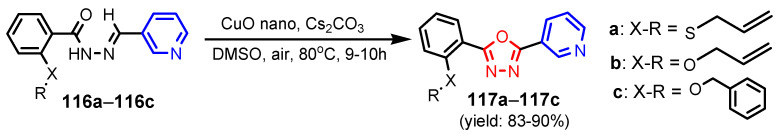
Synthesis of 1,3,4-oxadiazoles–pyridines **117a**–**117c** [54].

Murty et al. [54] synthesized 1,3,4-oxadiazoles–pyridines **117a**–**117c** through an oxidative C–O coupling by direct C–H bond activation of N-aroyl-N-arylidinehydrazines **116a**–**116c** using a catalytic quantity of CuO nanoparticles (Scheme 25). Compound **117a** showed broad-spectrum antibacterial activity for all tested strains, *E. coli*, *B. subtilis, M. luteus, K*. *pnemoniae* MIC 37.5 μg mL^−1^, and compounds **117a** and **117b** exerted antifungal activity better than standard drug, Cycloheximide.

**Scheme 26:**
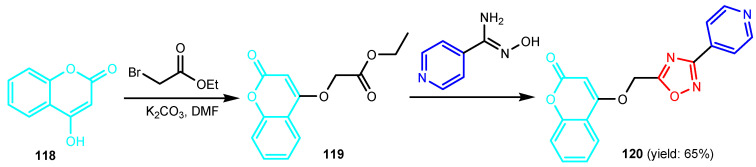
Synthesis of 4-[(3-(4-pyridyl)-1,2,4-oxadiazol-5-yl)methoxy]-coumarin **120** [55].

**Figure 12:**
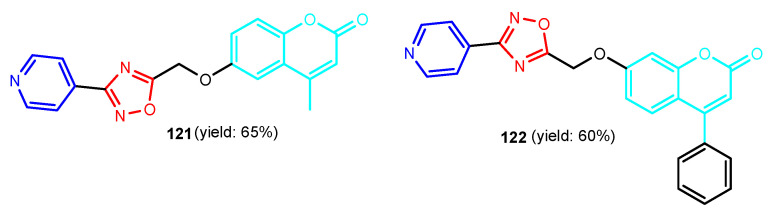
Structure of 1,2,4-oxadiazol-coumarin-pyridines **121** and **122**.

**Scheme 27:**
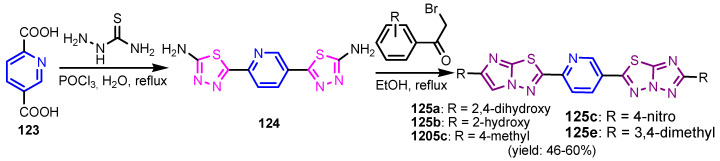
Synthesis of imidazo[2,1b]-1,3,4-thiadiazoles **120a**–**120e** [56].

**Scheme 28:**
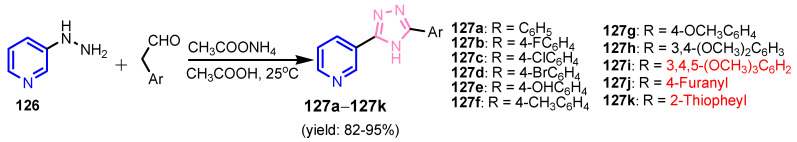
Synthesis of pyridine-triazoles **127a**–**127k** [57].

**Figure 13:**
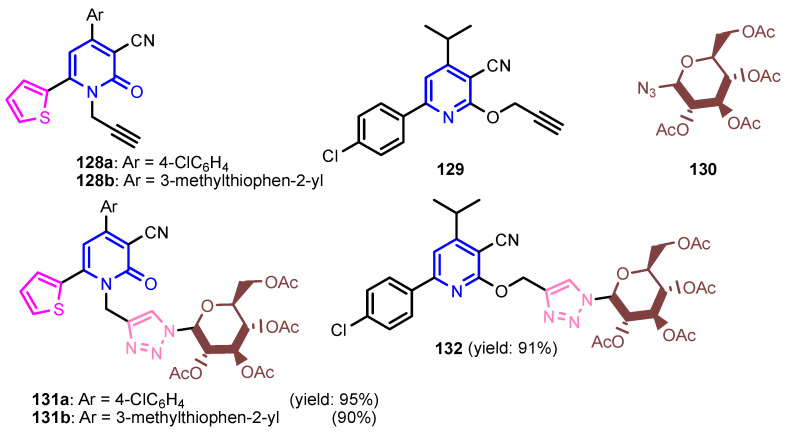
Structures of compounds **128**–**132** [58].

Krishna et al. [55] synthesized compound **120** in two steps: alkylation of 4-hydroxy-2H-chromen-2-one **118** to give ester **119**, which by condensation reaction with (Z)-N’-hydroxyisonicotinimidamide under reflux temperature for 24 h, gave 4-[(3-(4-pyridyl)-1,2,4-oxadiazol-5-yl)methoxy]-coumarin **120** (Scheme 26). Compounds **121** and **122** were synthesized by similar reactions (Figure 12) and showed good antibacterial activity against *S. aureus* and *E. coli*, and *pyridines* **120**–**122** presented better antifungal activity than that of standard drug, Clotrimazole. Bhardwaj et al. [56] synthesized pyridine imidazo [2,1b]-1,3,4-thiadiazoles **125a**–**125e** in two steps, from pyridine-2,5-dicarboxylic acid **123**, through intermediate **124** (Scheme 27). All compounds were found to be promising candidates against tested strains, *B. pumillus, S. aureus, V. cholera*, *E. coli*, *P. mirabilis*, *P. aeruginosa* and *C. albicans*. Panda and Jain [57] synthesized pyridine-triazoles **127a**–**127k** by a condensation reaction between 3-hydrazinylpyridine **126**, the corresponding aldehyde and ammonium acetate in acetic acid at 25 °C (Scheme 28). Minimum inhibitory concentrations (MIC, Table 6) showed excellent antimicrobial activity of compounds **127i**–**127k**, substituted with trimethoxyphenyl, furanyl and thiophenyl, as well as good activity of compounds **127b**, **127d**, **127g** and **127h**. Table 3 marked green the values identical to those of the standard medicines used, Ciprofloxacin and Miconazole. Morsy et al. [58] synthesized 1,2,3-triazole nucleosides based on functionalized nicotinonitriles **131a**–**131b** and **132**, via [2 + 3]-dipolar-cycloaddition of terminal acetylenes **128a**–**128b**, **129** and 1-azidoglucose **130** (Figure 13) in the presence of copper (I) ions generated from copper(II) sulfate and sodium L-(+)-ascorbate. Compounds **131a**–**131b** and **132** showed good in vitro antibacterial activity against *Staphylococcus aureus* ATCC 6538, *Micrococci luteus* ATCC 10240, *Pseudomonas aeruginosa* ATCC 9027, *Escherichia coli* ATCC 10536, *Candida albicans* ATCC 10231, and *Aspergillus niger* ATCC 16404.

## 6. Synthesis of Antimicrobial Pyridine Compounds Containing a Six-Membered Ring with One, Two, or Three Heteroatoms or Macrocycles

**Scheme 29:**
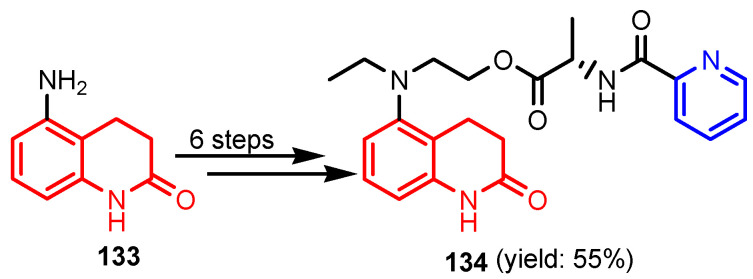
Synthesis of tetrahydroquinolin-pyridine **134** [59].

**Scheme 30:**
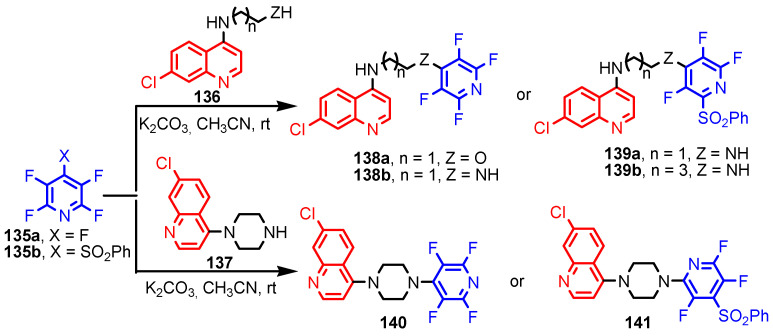
Synthesis of quinoline-pyridine compounds **136**–**141** [60].

**Scheme 31:**
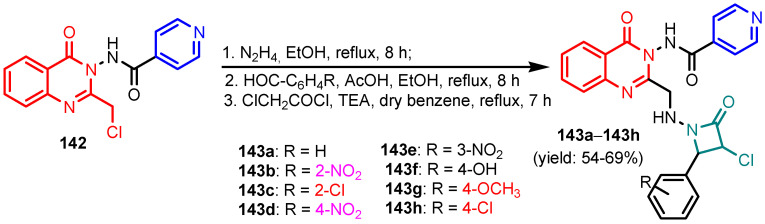
Synthesis of azetidinyl-3-quinazolin-4-one hybrids **143a**–**143h** [61].

**Scheme 32:**
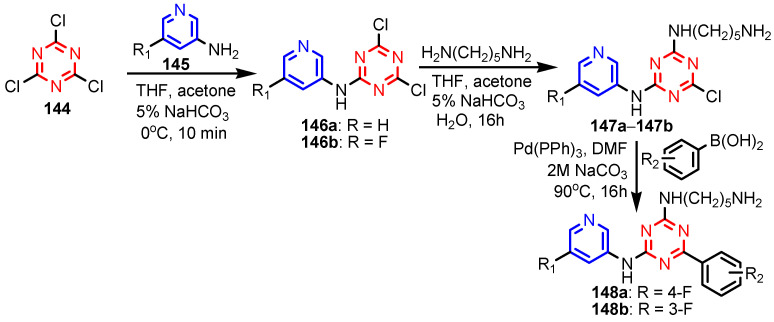
Synthesis of pyridine-fluorophenyl[1,3,5]triazines **143a**–**143b** [62].

**Figure 14:**
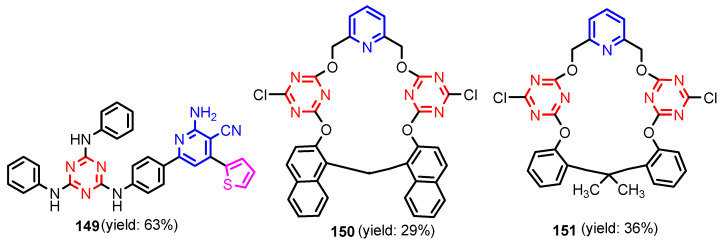
Structures of compounds **144–146** [63,64,65].

**Figure 15:**
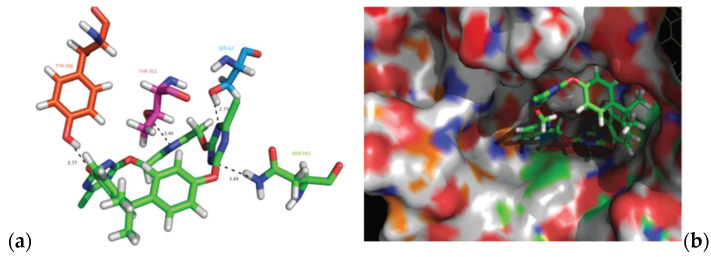
(**a**) Binding of oxacalixarene **151** with the covalent acyl enzyme (PDB ID: 1PW8). (**b**) The oxacalixarene substrate **151** bound to the binding site of quadruple mutant, showing the fitting of the test oxacalixarene on the substrate surface.

Deng et al. [59] synthesized (S)-2-(ethyl(2-oxo-1,2,3,4-tetrahydroquinolin-5-yl)amino) ethyl-2-(picolinamido)propanoate **134** in six steps, from 5-amino-3,4-dihydroquinolin- 2(1H)-one **133** (Scheme 29). Compound **134** showed moderated activity gainst all tested strains, *S. aureus*, *MRSA*, *B. subtilis*, *B. proteus*, *E. coli*, *P. aeruginosa*, *C. albicans*, *C. mycoderma*, *S. cerevisiae*, *C. neofonmans*, and *A. flavus*, considering Streptomycin and Fluconazole as standards. Ranjbar-Karimi and Poorfreidoni [60] reported the reaction of pentafluoropyridine **135a** with 2-((7-chloroquinolin-4-yl) amino)ethan-1-ol **136** in the presence of potassium carbonate in CH_3_CN, to give 7-chloro-N-(2-((perfluoropyridin -4-yl)oxy)ethyl)-quinolin-4-amine **138a**. Moreover, the reaction of N^1^-(7-chloroquinolin-4-yl) ethane-1,2-diamine **136** with 2,3,5,6-tetrafluoro-4-phenylsulphonyl pyridine **135b** in the presence of potassium carbonate in CH_3_CN, gave N^1^-(7-chloroquinolin-4-yl)-N^2^- (3,5,6-trifluoro-4-(phenylsulfonyl)pyridin-2-yl)ethane-1,2-diamine **139a** in a high yield. Similar reactions occurred to give products **138b**, **139b**, **140**, and **141**, respectively in good yields (75–80%) (Scheme 30). Compounds **138a**, **138b**, **139a**, and **141** showed moderate antibacterial activity against Gram-positive bacterium, *S. aureus*. Myangar and Raval reported [61] synthesis of azetidinyl-3-quinazolin-4-one hybrids **143a**–**143h** from N-(2-(chloromethyl)-4-oxoquinazolin-3(4H)-yl)isonicotinamide **142** in three steps: (1) synthesis of intermediate hydrazides by reflux with hydrazine in ethanol; (2) synthesis of Schiff bases by condensation of hydrazides with aldehydes in ethanol and acetic acid; and (3) synthesis of final azetidines **143** by reaction of Shiff bases with chloracetylchloride in dry benzene, in the presence of triethylamine (TEA) (Scheme 31). The antibacterial activity data indicate that the analogs with halogen and nitro substituents emerged as promising antimicrobials, showing better to moderate activity, while analogs bearing chloro and methoxy substituent showed better antifungal activities, see bold compounds (Table 7). Saleh et al. [62] synthesized pyridine-fluorophenyl[1,3,5]triazines **148a**–**148b** starting from 2,4,6-trichloro -1,3,5-triazine **144**, and pyridin-3-amines **145**, via intermediates **146** and **147**, and by using in the last step a Suzuki coupling reaction (Scheme 32). Compounds **148a** and **148b** showed excellent antibacterial activity against *S. epidermidis*, with MIC values of 16 μg mL^−1^ and 32 μg mL^−1^, respectively, taking Streptomycin sulfate as standard (MIC > 16 μg mL^−1^). Solankee and co-workers reported [63] that compound **149** (Figure 14) exhibited promising antibacterial activity against *Micrococcus flavus* with a zone of inhibition value (iv) of 7 mm, which was slightly less active than the standard Ampicillin (iv = 8mm) [54]. Rajavelu and Rajkumar reported [64,65] synthesis of triazino- oxacalixarenes **150** and **151** and their good antibacterial activity against E. coli (MIC = 50 μgmL^−1^). These oxacalixarenes were docked against the covalent acyl enzyme PDB: 1PW8, and the most potent compound **151** exhibited a good docking score, glide energy, a strong hydrogen bond and hydrophobic interactions with the amino acids in the active pocket. Moreover, oxacalixarene **151** showed strong hydrogen bond interactions with Ser 62, Asn 161, Thr301, and Tyr306 and hydrophobic interactions with Thr116, Phe 120, Thr123, Tyr159, Trp 233, Arg 285, Gln 303, and Asn 327, as seen in Figure 15. The computational work (molecular docking) for the oxacalixarenes used the Schrodinger software version 9.0 for the molecular docking.

## 7. Synthesis of Antimicrobial Macrocyclic Pyridine Compounds

**Scheme 33:**
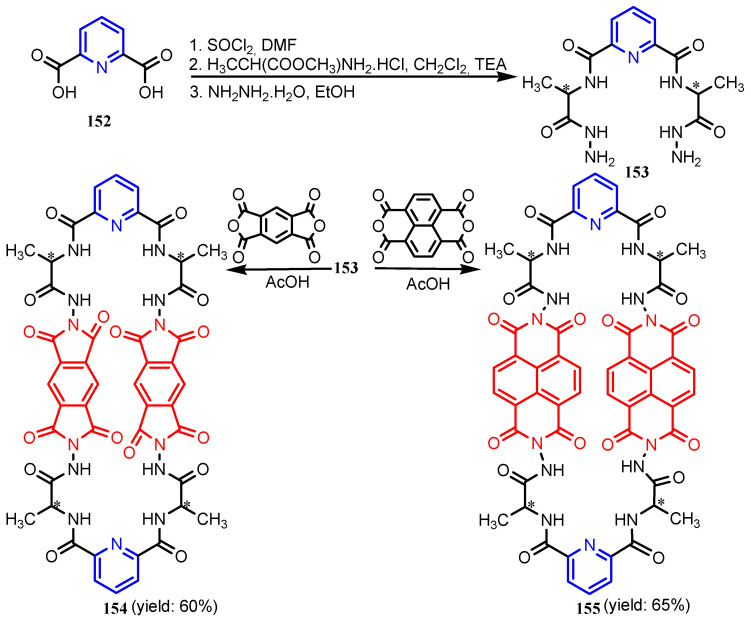
Syntheses of pyridine macrocyclic pyridine compounds **154** and **155** [66].

**Figure 16:**
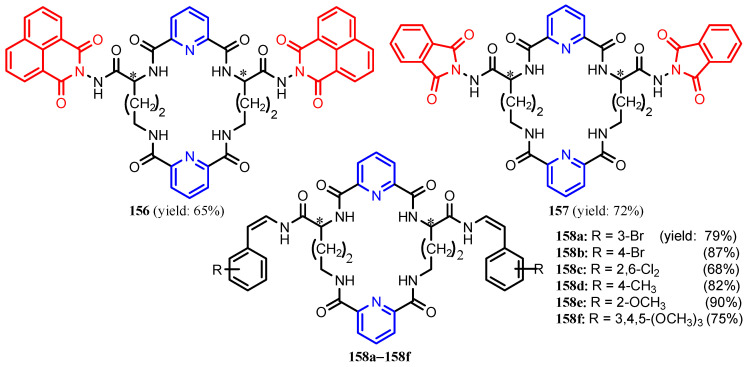
Structures of compounds **151**, **152** and **153a**–**153f** [67].

Al-Salahi et al. [66] synthesized N^2^,N^6^-bis(1-hydrazinyl-1-oxopropan-2-yl)pyridine-2,6-dicarboxamide **153**, in three steps from pyridine-2,6-dicarboxylic acid **152**. Reaction of **153** with 1,2,4,5-benzenetetracarboxylic acid dianhydride or 1,4,5,8-naphthalene tetracarboxylic acid dianhydride in refluxing acetic acid afforded the corresponding macrocyclic octaamide-tetraimide chiral pyridine derivatives **154** and **155**, respectively (Scheme 33). Table 8 shows that compounds **154** and **155** had significant antimicrobial activities against all tested strains, *B. subtilis*, *S. aureus*, *E. coli*, and *C. albicans*. Using a similar synthesis strategy, Abd El-Salam et al. [67] synthesized chiral macrocyclic polyamides **156**, **157** and **158a**–**158f** (Figure 16). As can be seen in Table 9, except for compounds **158b**, **158d** (against *B. subtilis*) and **158c** and **158e** (against *S. aureus*), the activities of the tested compounds against the Gram-positive bacteria are slightly higher than that of the reference drug Ampicillin. Compounds **156** and **158a** also had better inhibitory activity than Ampicillin against *E. coli*. The antifungal activity of compounds **156**–**158** was moderate considering the standard drug Ketoconazole.

## 8. Synthesis of Fused Pyridines with Other Heterocycles with Antimicrobial Properties

**Scheme 34:**
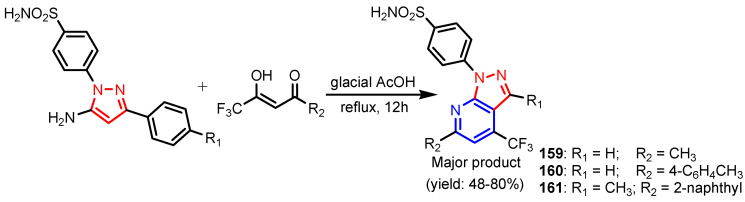
Synthesis of pyrazolo[3,4-b]pyridines **159**–**161** [68].

**Scheme 35:**
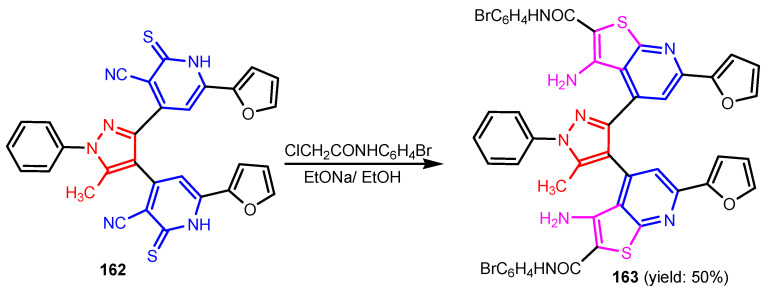
Synthesis of thieno[2,3-b]pyridine **163** [69].

**Scheme 36:**
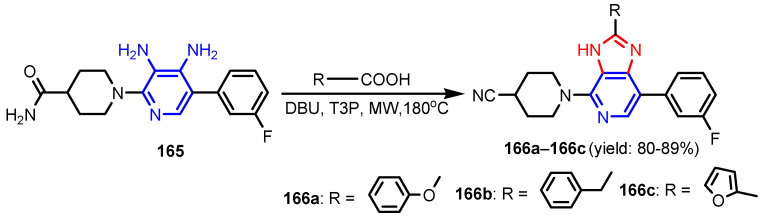
Synthesis of imidazo[4,5-c]pyridines **166a**–**166c** [70].

**Figure 17:**
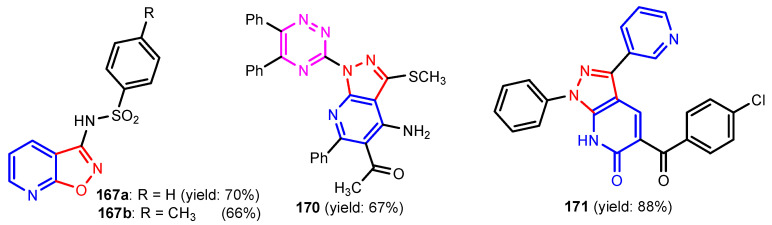
Structures of compounds **167a**, **167b**, **170**, and **171** [71,73,74].

**Scheme 37:**
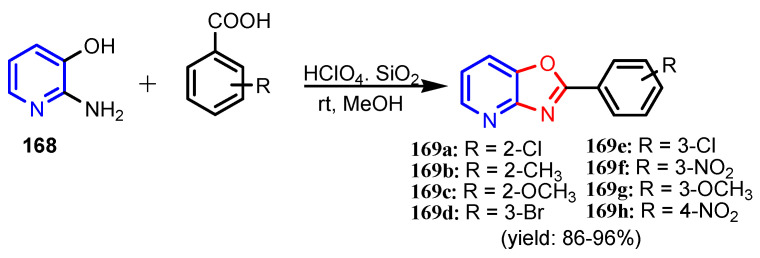
Synthesis of oxazolo[4,5-b]pyridines **169a**–**169h** [72].

**Scheme 38:**
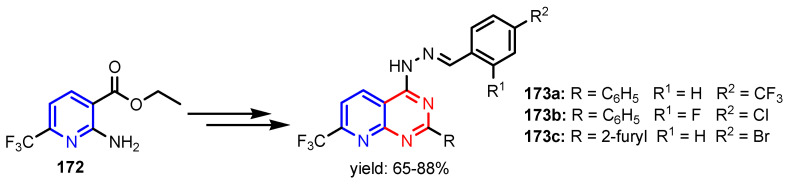
Synthesis of pyrido[2,3-d]pyrimidines **173a**–**173c** [75].

**Scheme 39:**
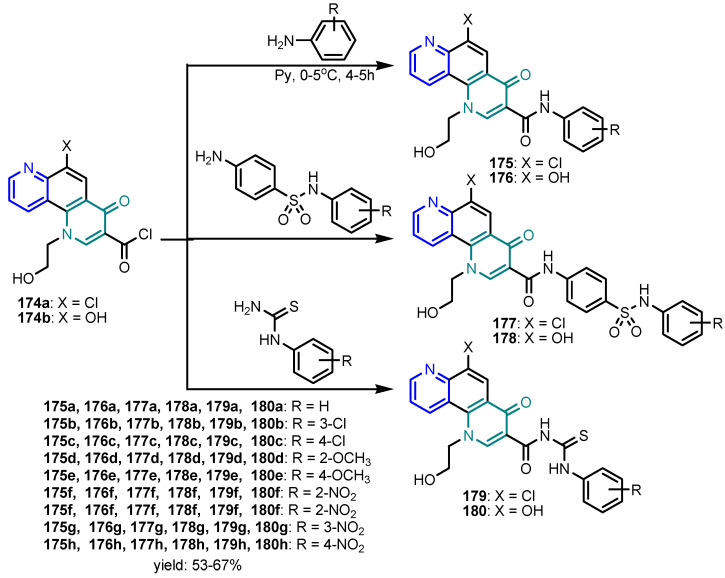
Synthesis of pyridoquinolone **175**–**180** [76].

Chandak et al. [68] synthesized pyrazolo[3,4-b]pyridines **159**–**161** by reaction between 3-substituted-(5-amino-1*H*-pyrazol-1-yl)benzenesulfonamide and appropriate trifluoro methyl-β-diketone (Scheme 34). Compound **159** was found to be the most potent analogue exhibiting a moderate antibacterial activity against Gram-positive bacteria, *S. aureus* and *B. subtilis*, while **160** and **161** exhibited moderate antifungal activity against *S. cerevisiae* and *C. albicans*, respectively (Table 10). Altalbawy [69] reported a reaction between compound **162** with 2-chloro-N-(4-bromophenyl) acetamide, in ethanolic sodium ethoxide solution at room temperature to give 4,4′-(5-methyl-1-phenyl-1*H*-pyrazole-3,4-diyl)bis-6-(2-furyl) thieno[2,3-b]pyridine-2-carboxamide **163** (Scheme 35). Compound **163** had moderate antimicrobial activity against *E. coli* and *C. albicans* strains. Jose et al. [70] synthesized imidazo[4,5-c] pyridines **166a**–**166c** from substituted 3,4-diamino pyridine and carboxylic acids in the presence of DBU (1,8-Diazabicyclo(5.4.0)undec-7-ene) mediated by T3P (anhydride of propanephosphonic acid) (Scheme 36). All compounds displayed a promising antimicrobial activity compared to control antibiotic, Streptomycin and antifungal drug, Fluconazole. Poreba et al. [71] reported synthesis of new sulfonamide isoxazolo[5,4-b]pyridines **167a**–**167b** by reaction between aryl sulfonic chlorides and amines (Figure 17). Both compounds showed moderate antibacterial activity against *E. coli* and *P. aeruginosa*. Reen et al. [72] prepared 2-(substituted-phenyl)oxazolo[4,5-b]pyridines **169a**–**169h** using a one pot synthetic strategy in quantitative yield by reaction between 2-amino-3-hydroxy pyridine **168** and substituted benzoic acids in the presence of silica-supported perchloric acid (Scheme 37). As can be seen in Table 11, three compounds (**169c**, **169f**, **169g**) possessed strong antibacterial activity against all the four strains, with the best activity profile against *S. aureus*, and the other five compounds were found to be moderately active against all the tested strains of bacteria. Ali [73] reported synthesis of pyrazolo[3,4-b]pyridine **170** by treatment of 5-amino-1-(5,6-diphenyl-1,2,4-triazin-3-yl)-3-(methylthio)-1*H*-pyrazole-4-carbonitrile with benzoyl acetone in basic medium (Figure 16). Compound **170** showed moderate antimicrobial activity against tested strains, *S. ureus*, *S. epidermidis*, *E. coli*, and *A. fumigatus.* El-Borai et al. [74] reported that compound 5-(4-chlorobenzoyl)-1-phenyl-3-(pyridin-3-yl)-1*H*-pyrazolo[3,4-b]pyridin-6(7*H*)-one **171** exhibited strong activity against *E. coli*, *E. cloaca*, and *Serratia.* Appna et al. [75] synthesized pyrido[2,3-d]pyrimidines **173a**–**173c** in four steps from ethyl 2-amino-6-(trifluoromethyl) nicotinate **172** (Scheme 38). All compounds exhibited significant antifungal activities against tested strains, *C. albicans*, *C. parapsilosis*, *C. glabrata*, *C. krusei*, and *Issatchenkia hanoiensis.* Patel et al. [76] reported synthesis of pyridoquinolones **175–180** from substituted aniline, substituted phenyl thioureas and 4-amino-N-(substitutedphenyl) benzenesulfonamide (Scheme 39). The structure–activity relationship study for pyridoquinolones demonstrated that both electron withdrawing as well as eletrodonating groups at the phenyl ring increase the antimicrobial activity. Furthermore, the presence of chloro and hydroxyl groups at the C–6 position showed a similar activity. Activities of amides increased in order: thioureido amides < sulfonamides < amides. Antibacterial activities increased in order: *P. aeruginosa* < *S. aureus* < *B. substilis* < *E. coli*.

**Figure 18:**
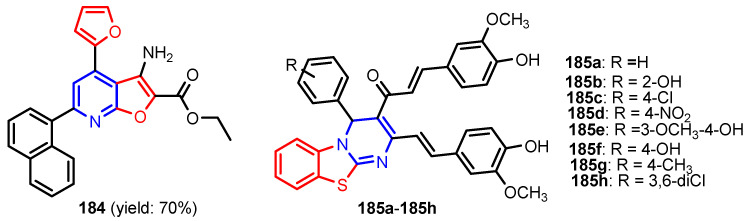
Structures of compounds **184** and **185a**–**185h** [78].

**Scheme 40:**
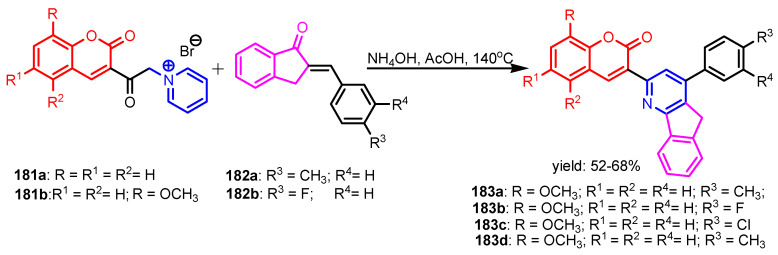
Synthesis of pyridoquinolone **183a**–**183b** [77].

**Figure 19:**
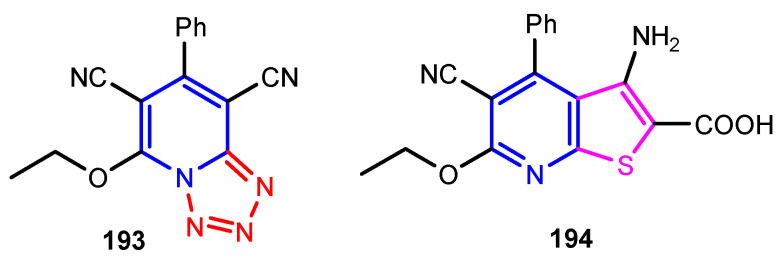
Structures of compounds **193** and **194** [81].

**Scheme 41:**
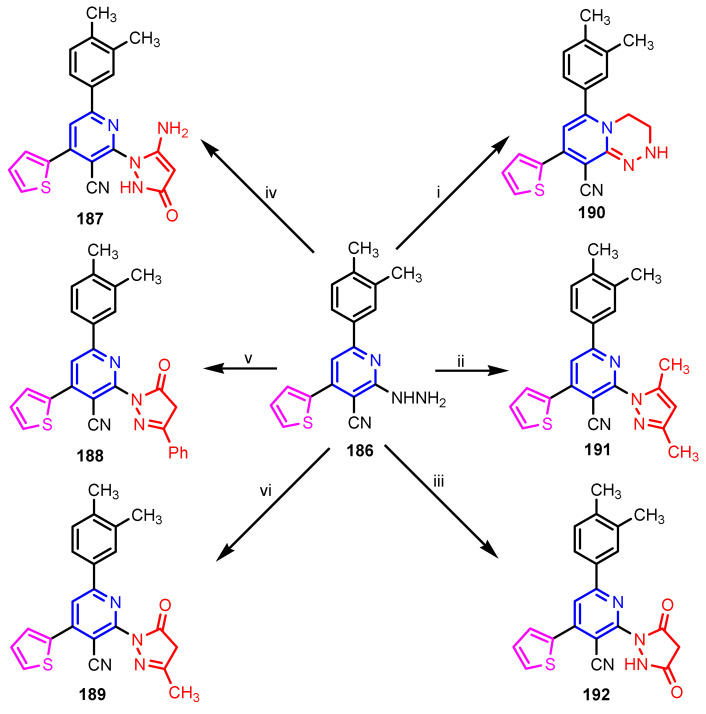
Synthesis of compounds **187**–**192**; reagent and conditions: (i) ClCH_2_CH_2_Cl, DMF, heat; (ii) CH_3_COCH_2_COCH_3_, EtOH, heat; (iii) CH_2_(COOEt)_2_, EtOH, heat; (iv) NCCH_2_CO_2_Et, EtOH, heat; (v) PhCOCH_2_CO_2_Et, EtOH, heat; (vi) 1-CH_3_COCH_2_CO_2_Et, EtOH, heat; 2- EtONa, heat. [80].

Brahmbhatt et al. [77] synthesized coumarin-indenopyridine **183** using Krohnke’s conditions (Scheme 40), by the reaction of 3-coumarinylmethylpyridinium salts **181a**–**181b** with appropriate 2-arylidene-1-indanones **182a**–**182b**. Compounds **183a** and **183b** were the most potent against *S. aureus* and *B. subtilis*, respectively, while the rest of the compounds have moderate antibacterial and antifungal activity considering Ampicillin as standard. Salem et al. [78] reported the synthesis of ethyl 3-amino-4-(furan-2-yl)-6-(naphthalen-1-yl)furo[2,3-b]pyridine-2-carboxylate **184** using a one-pot four-component reaction. Compound **184** has a moderate antibacterial activity against *E. coli* and *S. aureus* considering Tetracycline as standard (Figure 18). Youssoufi et al. [79] reported the synthesis of 4*H*-pyrimido[2,1-b]benzothiazoles **185a**–**185h** from curcumin and their antimicrobial activities against *S. aureus*, *S. typhi*, *P. aeruginosa*, *E. coli*, *B. cereus*, and *P. rettgeri* bacteria. All compounds showed moderate to high antibacterial activities (Table 12). Flefel et al. [80] reported the synthesis of compounds **187–192** in good yields (51–79%) from pyridine **186** using the pathways showed in Scheme 41. All synthesized compounds showed moderate to good antibacterial and antifungal activities against tested strains, *S. aureus*, *B. subtilis*, *E. coli*, *S.* *typhimurium*, *A. flavus*, and *C. albicans*, as compared to standard reference drugs, Gentamycin and Ketoconazole. El-Sayed et al. [81] reported that tetrazolo[1,5-a]pyridine derivative **193** and thienopyridine **194,** synthesized from 6-ethoxy-2-oxo-4-phenyl-1,2-dihydropyridine-3,5-dicarbonitrile, have good antimicrobial activities against *S. aureus*, *B. cereus*, *E. coli*, *P. aeruginosa*, *A. flavus*, and *A. niger* as compared to standard drugs, Cefotaxime and Dermatin (Figure 19).

**Scheme 42:**
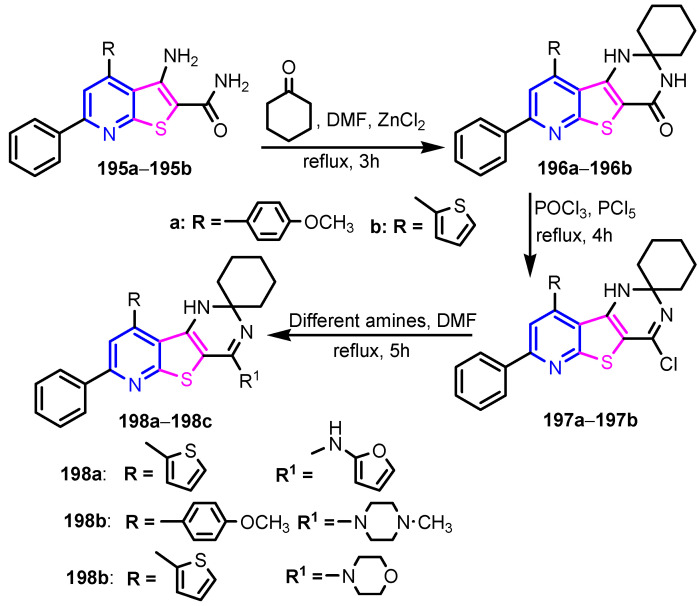
Synthesis of compounds **196**–**198** [82].

El-Deen et al. [82] reported the synthesis of the pyridothienopyrimidine derivatives **196**–**198** in good yields (70–81%) from compounds **195a**–**195b**, as can be seen in Scheme 42. The in vitro evaluation of these compounds against six strains of both Gram-positive (*S. aureus*, *B. Subtilis*, *B. cereus*) and Gram-negative (*E. coli*, *S. typhimurium*, *P. aeruginosa*) bacteria compared with Amoxicillin trihydrate as a reference drug showed a significant antibacterial activity for all compounds, especially against Gram-negative strains, with MIC values ranging from 7.81 to 62.5 μg mL^−1^

## 9. Synthesis of Pyridine Compounds Containing P, Se, and B with Antimicrobial Properties

**Scheme 43:**
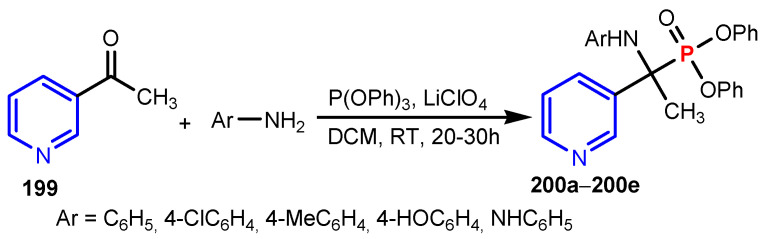
Synthesis of phosphorus containing pyridines **200a**–**200e** [83].

Abdel-Megeed et al. [83] synthesized a series of diphenyl 1-(arylamino)-1-(pyridin-3-yl) ethyl phosphonates **200a**–**200e** in high yields from reactions of 3-acetyl pyridine **199** with aromatic amines and triphenylphosphite in the presence of lithium perchlorate as a catalyst (Scheme 43). All compounds showed high antimicrobial activities against *Escherichia coli* NCIM2065, *Bacillus subtilis* PC1219, *Staphylococcus aureus* ATCC25292, *Candida albicans*, and *Saccharomyces cerevisiae*, at low concentrations (10–100 μg mL^−1^, Table 13).

**Scheme 44:**
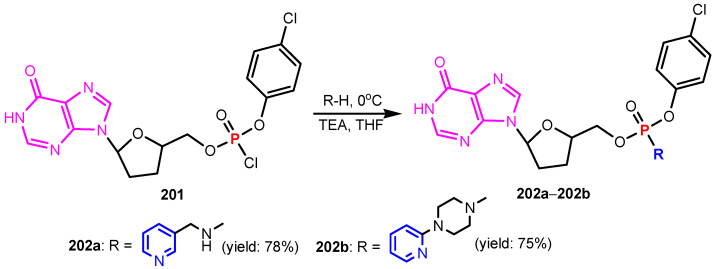
Synthesis of phosphorus containing pyridines **202a**–**202b** [84].

**Scheme 45:**
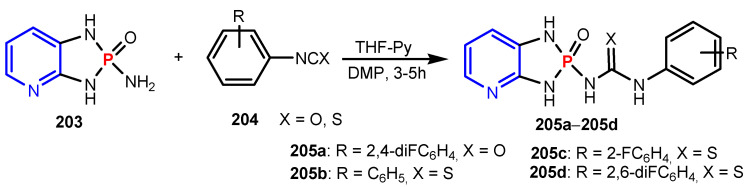
Synthesis of phosphorus containing pyridines **205a**–**205d** [85].

Sekhar et al. [84] synthesized phosphorylated nucleosides **202a**–**202b** from the reaction between compound **201** and the corresponding pyridines (Scheme 44). Both compounds **202a**–**202b** exhibited better activity on *Staphylococcus aureus* when compared to standard, Gentamycine. Devineni et al. [85] synthesized compounds **205a**–**205d** by reaction of 2-amino-2,3-dihydro-1*H*-2λ^5^-[1,3,2]diazaphospholo[4,5-b]pyridin-2-one **203** and compounds **204** (Scheme 45). Urea derivatives **205a**, **205b**, **205c**, and **205d** showed growth of inhibition against all the tested bacterial strains *B. cereus* ATCC 11778, *S. faecalis* MTCC 0459, *E. coli* ATCC 9637, and *P. marginalis* MTCC-2758.

**Scheme 46:**
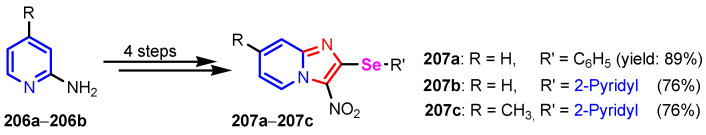
Synthesis of selenium containing pyridines **207a**–**207c** [86].

Kumar et al. [86] synthesized organoselenium imidazo[1,2-a]pyridines compounds **207a**–**207c** in four steps, starting with 2-aminopyridines **206a**–**206b** (Scheme 46). Compounds **207a** and **207c** were observed to exhibit antibacterial activity with an MIC value of 2.48 μg mL^−1^ and 10.41 μg mL^−1^ respectively, against Gram-negative bacteria *E. coli*. These observed activities, in particular the one observed for compound **207a** against *E. coli*, have been found to be more potent as compared to the few reports concerning antimicrobial activity of selenide-based compounds in the literature so far and quite similar to the Rifampicin (standard). Compound **207b** was found to be exhibiting multi-spectrum activity against *A. fumigates*, *C. krusei*, *A. niger*, and *C. parapsilosis* with observed MIC values of 9.96, 9.96, 19.93, and 19.93 μg mL^−1^ respectively.

**Scheme 47:**
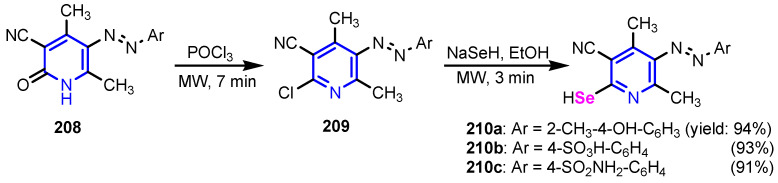
Synthesis of selenium-containing pyridines **210a**–**210c** [87].

**Figure 20:**
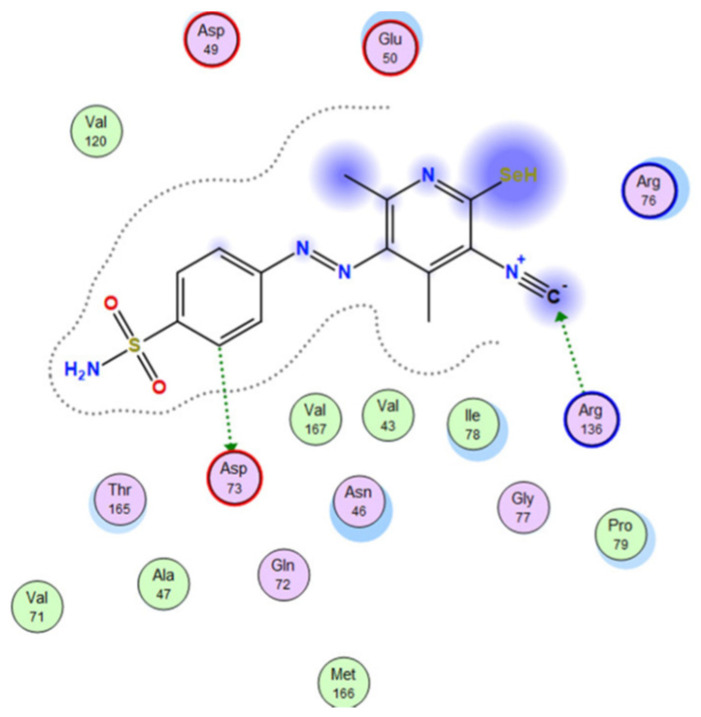
3D interaction of selenium compound **210c** with 1kzn protein [87].

**Scheme 48:**
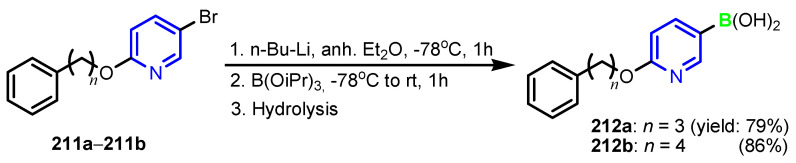
Synthesis of boron containing pyridines **210a**–**210c** [88].

Abdellattif et al. [87] synthesized organo-selenium compounds **210a**–**210c** starting from 4,6-dimethyl-2-oxo-1,2-dihydropyridine-3-carbonitrile **208**, in two steps (Scheme 47). Selanone **210c** was found to be the most potent antibacterial agent against all tested strains, *S. aureus*, *S. pyogenes*, *E. coli*, and *P. aeruginosa*, as well as a good antifungal agent, against *C. albicans*, *A. niger*, and *A. clavatus*. A computational study of selenium compounds was performed for prediction of ADME properties by the QikProp3.2 tool available in Schrödinger 9.0 version (USA) and Molinspiration online property calculation toolkit to understand whether the compounds have the optimum pharmacokinetic properties to enter higher phases of the drug development process or not. It was found that compound **210c** displayed the highest energy interaction (−8.48 KcalMol^−1^) within the binding pocket in the molecular docking study, as can be seen in Figure 20. Fontaine et al. [88] reported the synthesis of the boronic pyridines **212a** and **212b** (Scheme 48), which showed a 4-fold increase in activity compared to 6-(benzyloxy)pyridin-3-ylboronic acid. Furthermore, they emerged as potential Staphylococcus aureus NorA inhibitors as they potentiate the activity of Ciprofloxacin and Norfloxacin, have no significant intrinsic antibacterial activity, show a reduced cytotoxicity, and do not inhibit the mammalian P-gp efflux pump.

## 10. Synthesis of Antitubercular Pyridine Compounds

Tuberculosis (TB) remains a major threat to global public health, with at least 10 million new cases and 1.2 million deaths in 2019. The high incidence and mortality rates of TB can be attributed to the capacity of its etiological agent, intracellular bacterium Mycobacterium tuberculosis (MTb), to adapt to and survive in the aggressive physiological environment within the host [89]. The need for new tuberculostatic agents arises due to bacterial resistance to first-line drugs: Isoniazid (INH), Rifampicin (RIF), Ethambutol (ETH), Streptomycin (STR), and Pyrazinamide (PYR).

**Scheme 49:**
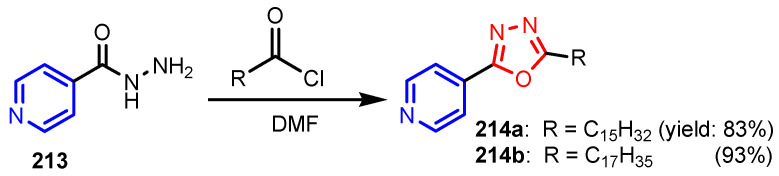
Synthesis of (1,3,4-oxadiazol-2-yl)pyridines **214a**–**214b** [90].

Navarrete-Vázquez et al. [90] synthesized 4-(5-substituted-1,3,4-oxadiazol-2-yl)pyridines **214a** and **214b** from INH and acid chlorides (Scheme 49). Compound **214a** showed 10 and 20 times more potency than INH and STR, respectively, against the INH-resistant *M. tuberculosis* CIBIN 112 strain. Moreover, this compound was 27 times more active than ETH. Compound **214b** showed the same behavior as **214a** against this strain.

**Figure 21:**
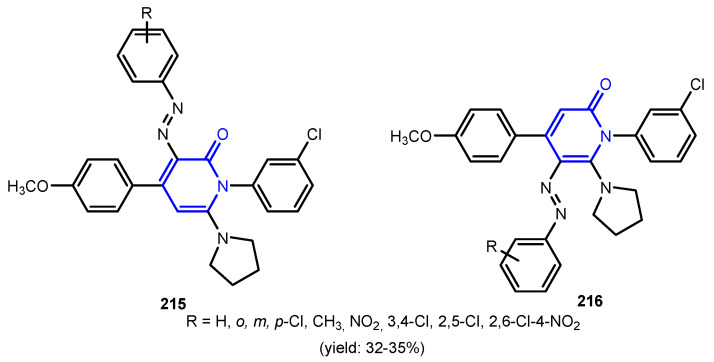
Structures of compounds **215** and **216**.

**Scheme 50:**
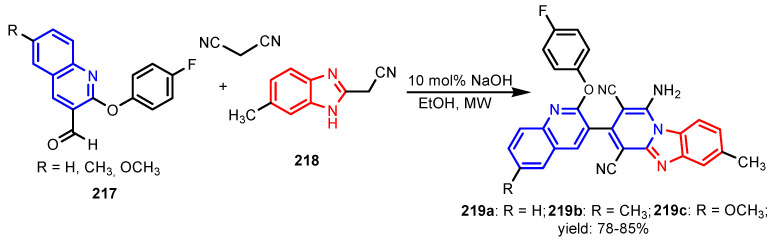
Synthesis of pyrido[1,2-a]benzimidazole derivatives **219a**–**219c** [92].

Patel et al. [91] reported two series of compounds **215** and **216** with poor activity against *M. tuberculosis* when compared with Rifampicin (Figure 21). Furthermore, compounds showed a very good activity against *C. albicans* and possessed moderate to poor activity against *A. niger* and *A. clavatus* when compared to the standard, Griseofulvin. Sangani et al. [92] reported the synthesis of pyrido[1,2-a]benzimidazole derivatives **219a**–**219c**, via a base-catalyzed microwave-assisted multicomponent cyclocondensation reaction (Scheme 50). Evaluation of antitubercular activity shows that compounds **219b** and **219c** were found to have better antitubercular activity when compared to standards Isoniazid and Rifampicin, and compound **219a** appeared as the promising antimicrobial compound with a significant antitubercular activity.

**Scheme 51:**
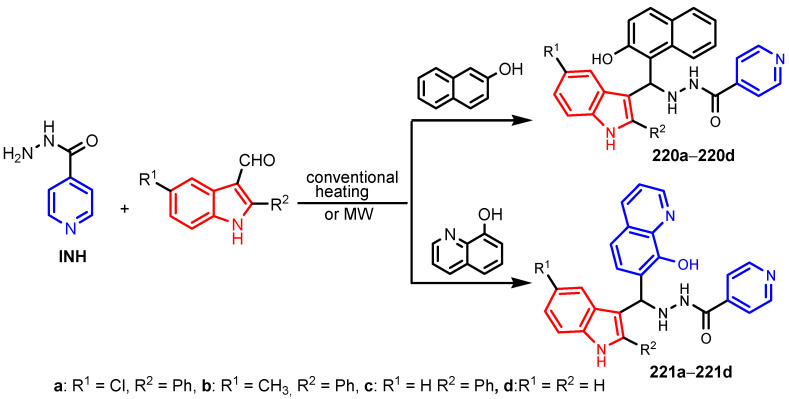
Synthesis of Isoniazid (INH) derivatives **220** and **221** as antitubercular agents [93].

Rathod et al. [93] synthesized a series of INH compounds **220a**–**220d** and **221a**–**221d** using multicomponent reactions in good yields (80–94%), as can be seen in Scheme 51. Electron-withdrawing groups were reported to increase the anti-TB activity [94,95]. Compound **220c** (R^1^ = H) showed an MIC value of 3.12 μg mL^−1^, the minimum inhibitory concentration of **220a** (R^1^ = Cl) was estimated at 6.25 μg mL^−1^, and the MIC value of **221a** (R^1^ = Cl) was 12.5 μg mL^−1^. The other compounds were moderately active (MIC 50 μg mL^−1^) against M. tuberculosis H37Rv in comparison to the standard antitubercular drugs isoniazid (INH), pyrazinamide (PZA), streptomycin (STM), and ciprofloxacin (CPF), as can be seen in Table 14.

## 11. Synthesis of Antiviral Pyridine Compounds

In recent years, several important viral infections have emerged and antiviral chemotherapeutic agents are not sufficiently effective in clinic, leading to serious human diseases and mortality. Therefore, novel antiviral candidates are urgently desirable, which is undoubtedly essential for the therapy of various fatal viral infections. Pyridine compounds are obtaining importance in the field of medicinal chemistry because of the broad spectrum of their physiological activities. In this part of the review is highlighted antiviral behavior of pyridine compounds [96]. The pandemic of coronavirus disease 2019 (COVID-19) caused by the novel severe acute respiratory syndrome coronavirus 2 (SARS-CoV-2) presents an unprecedented challenge to identify effective drugs for prevention and treatment [97].

**Figure 22:**
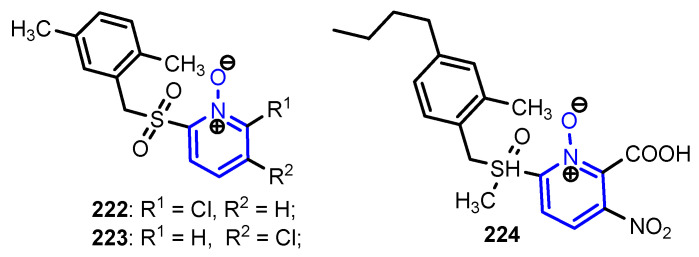
Structures of compounds **222**, **223**, and **224**.

Balzarini et al. [98] reported the synthesis of pyridine N-oxide derivatives and the inhibitory effect of these compounds on human SARS and feline infectious peritonitis coronavirus in cell culture. Thus, compounds **222** and **223** were the most interesting compounds that had a comparable (potent) activity against both SARS-CoV and feline coronavirus FIPV strain (EC_50_: 1.7–4.2 mg L^−1^), being poorly cytotoxic (Figure 22). Furthermore, they reported that a lack of the oxide moiety proved detrimental for anti-SARS-CoV and anti-FIPV activity, as none of the tested compounds was antivirally active at subtoxic concentrations [99].

**Figure 23:**
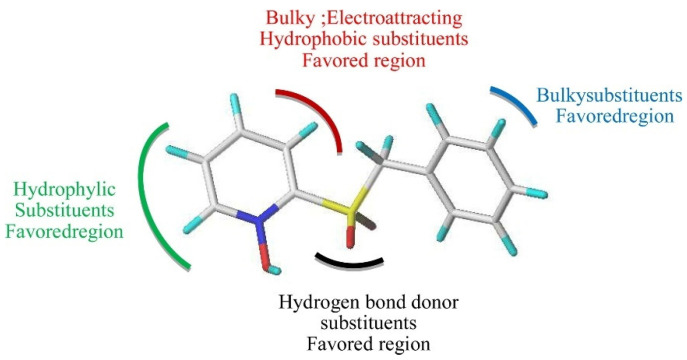
Different regions that can increase or decrease the antiviral activity [100].

Continuing their research, the Ghaleb group [100] found the structural conditions that N-oxides must meet in order to have an increased antiviral activity, which is shown in Figure 23. In this study, pyridine N-oxide **223** with SARS-CoV inhibitory activity was selected for a further 3 D-QSAR studies. From 3D QSAR, molecular docking modeling, it was found that compound **224** is more potent than chloroquine and hydroxychloroquine against SARS-CoV-2. Moreover, **223** shows an excellent stability to inhibit the main protease 3CLpro of CoV-2 and also has excellent bioavailability and no toxicity results. In this study, Surflex-Dock was used for molecular docking using SYBYL-X.2.0 software The crystal structure of COVID-19 main protease was downloaded from Protein Data Bank in Europe (PDB ID: 6LU7).

**Scheme 52:**
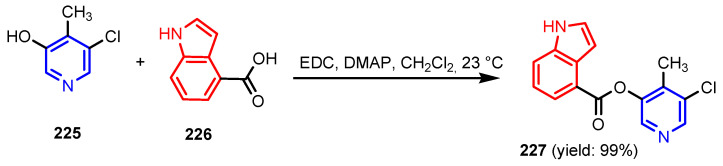
Synthesis of pyridine compound **227** [101].

**Figure 24:**
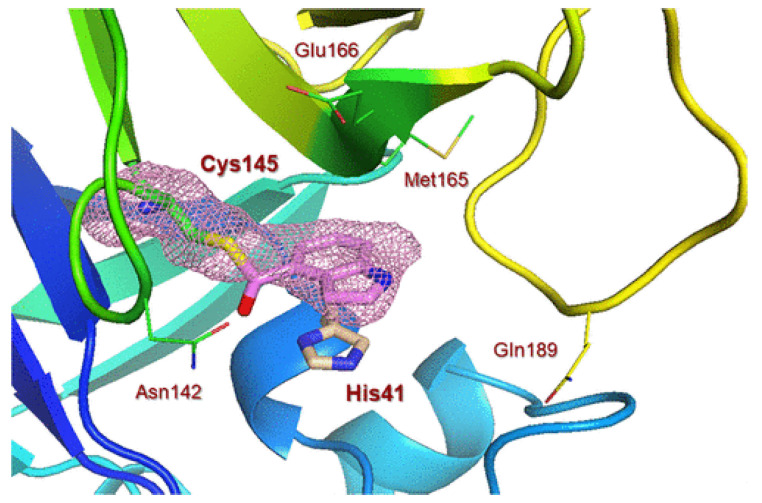
X-ray structure of SARS-CoV-2 3CLpro bound with compound **227** at 1.65 Å.

Ghosh et al. [101] reported synthesis of 5-chloro-4-methylpyridin-3-yl-1*H*-indole- 4-carboxylate **227** from 5-chloro-4-methylpyridin-3-ol **225** and 1H-indole-4-carboxylic acid **226** in the presence of EDC (1-ethyl-3-carbodiimide hydrochloride) and DMAP (4-dimethyl aminopyridine) using dichloromethane as solvent (Scheme 52). Compound **227** exhibited potent antiviral activity (EC_50_ ∼ 2.2 μM) against SARS-CoV-2 3CLpro, which was comparable to antiviral medicine Remdesivir. The authors demonstrate that the antiviral activity of a compound is not because of misleading cytostatic effects or cytotoxic effects but due to an apparent destructive “antiviral effect.” The determined high-resolution X-ray structures of compounds bound to SARS-CoV 3CLpro and SARS-CoV-2 3CLpro enzymes revealed that catalytic Cys145 formed a covalent bond with the indole carbonyl group. Furthermore, the indole rings of the inhibitor formed π−π stacking interactions with the imidazole ring of His41, a residue that must move substantially before, during, and after reaction with the inhibitors (Figure 24). X-ray data of the compounds were indexed, integrated, and scaled using the HKL2000 software package.

**Scheme 53:**
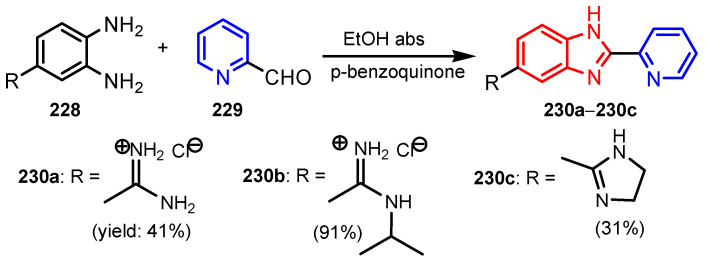
Synthesis of pyridine compounds **230a**–**230c** [102].

Starčević et al. [102] reported the synthesis of 2-substituted-5-amidino-benzimidazoles **230a**–**230c** from the reaction of 1,2-phenylenediamine **228** and picolinaldehyde **229**. All compounds possessed excellent antiviral activity against coxsackievirus B5 and echovirus 7, with EC_50_ values of 0.33–56.7 μM (Scheme 53, Table 15).

**Figure 25:**
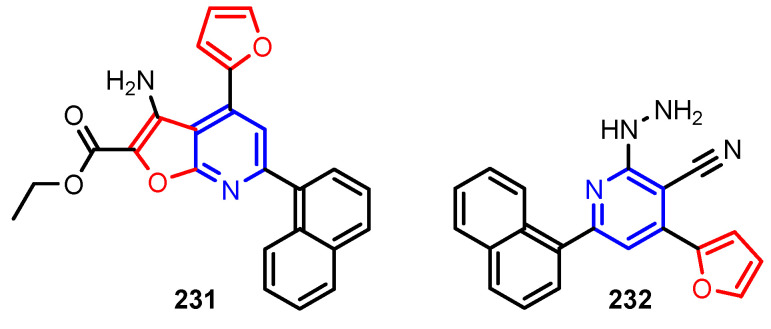
Structures of compounds **231** and **232** [103].

**Scheme 54:**
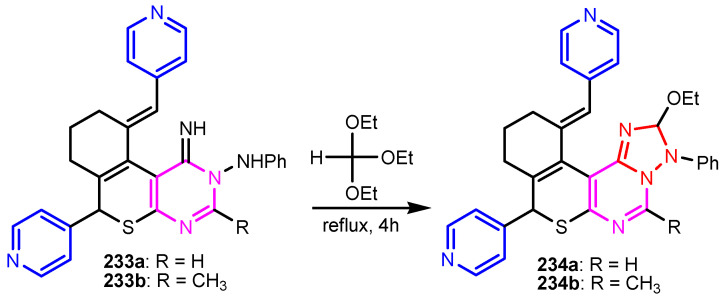
Synthesis of pyridine compounds **234a** and **234b** [104].

Salem et al. [103] reported the synthesis of compound **231** by cyclisation of ethyl 2-(3-cyano-4-(furan-2-yl)-6-(naphthalen-1-yl)pyridin-2-yloxy)acetate in the presence of alcoholic KOH 10% at reflux and compound **232** by refluxing 4-(furan-2-yl)-2- hydrazinyl-6-(naphthalen-1-yl) nicotinonitrile with POCl_3_ and PCl_5_, in a water bath (Figure 25). Compounds **231** and **232** showed antiviral activity against rotavirus Wa strain and adenovirus type 7. Compound **231** showed a 60% and 53.3% reduction in viral titer with rotavirus Wa strain and adenovirus type 7, respectively. Compound 232 showed 53.3% and 50% reduction with rotavirus Wa strain and adenovirus type 7, respectively. Sekhar et al. [84] reported that compounds **202a** and **202b** (Scheme 44) exhibited the better antiviral activities against Newcastle disease virus (NDV) in embryonated chicken eggs by vanishing of virus at the sixth titer (1/32). Abu-Hashem et al. [104] reported the synthesis of compounds **234a** and **234b** by reaction of **233a** and **233b**, respectively, with triethoxy-methane (Scheme 54). The data revealed that isothio chromeno-triazolopyrimidines **234a** and **234b** have promising in vitro antiviral activity against herpes simplex virus-1 (HSV-1) and human immunodeficiency virus-1 (HIV-1).

## 12. Conclusions

This review summarizes the syntheses of pyridine compounds with the antimicrobial and antiviral properties mentioned in the literature. Bioinformatics analysis plays a central role in finding new antimicrobial and antiviral structures, as is seen in the particular cases shown in the article. From the data presented, it can be concluded that the presence of another heterocycle (pyrazole, imidazole, thiazole, pyrimidine, benzimidazole, indole, etc.) on pyridine compounds improves antimicrobial or antiviral activities. In pyridinium salts it was found that an appropriate balance between hydrophobicity and hydrophilicity or an alkyl chain of C12–C16 improves the antimicrobial activity. Moreover, the presence of certain groups grafted on pyridine nuclei, such as -F, -Cl, -OH, -CF_3_, OCH_3_, -N(CH_3_)_2_, -NHCO, -COOCH_3_, -CHO, -NO_2_, and -CN, increases the antimicrobial and antiviral activity of the compounds. As is shown, the positions of the substituents on the pyridine molecule are also very important for the antimicrobial and antiviral activity of the compounds. Additionally, the binding linker between pyridine and another heterocycle is important for their antimicrobial and antiviral activity. Additionally, the antimicrobial activity is improved if the molecule contains linker groups such as carbonyl (CO), amide (NHCO), thioamide (NHCS), or other heteroatoms. It is also noted that pyridine compounds containing P, Se, and B, generally have an improved antimicrobial activity compared to those with only heterocyclic rings.

From the literature reports presented, we can highlight the following aspects related to the interaction of pyridine compounds, which determine the selectivity of antimicrobial action:

- a favorable binding interaction with Thymidylate kinase (ID: 4QG), within the binding pocket of TMPK, determines a good antimicrobial activity of bis (1*H*-indol-3-yl)-pyridine **78d**, against *S. aureus*, *E. coli*, and *C. albicans* strains;
- affinity and activity of thiophene-pyridines **82**–**84** on the target protein GlcN-6-P synthase is supported by in vitro antimicrobial screenings against almost all tested strains, *S. aureus*, *B. subtilis*, *E. coli*, *S. typhimurium*, *A. flavus*, and *C. albicans*;
- The good antimicrobial activity of triazino-oxacalixarenes **150** and **151** against *E. coli* is due to a good covalent interaction with acyl enzyme PDB: 1PW8;
- 3D interaction of selenium compound **210c** with 1kzn protein, the lower hydrophobicity, higher topological polar surface area, lower dipole moment, high H-bonding acceptor and donor, and in-silico absorption percentage of it explicates its highest antimicrobial activity against all tested strains, *S. aureus*, *S. pyogenes*, *E. coli*, *P. aeruginosa*, as well as a good antifungal agent, against *C. albicans*, *A. niger*, and *A. clavatus*;
- 3D QSAR, molecular docking modeling showed that pyridine-N-oxide **223** shows an excellent stability to inhibit the main protease 3CLpro of CoV-2 and also has excellent bioavailability and no toxicity results;
- the excellent antiviral activity of compound **227** (EC50∼2.2 μM) against SARS-CoV-2 3CLpro comparable to antiviral medicine Remdesivir is due to binding of the compound to SARS-CoV 3CLpro and SARS-CoV-2 3CLpro enzymes, which revealed that catalytic Cys145 formed a covalent bond with the indole carbonyl group, and also indole rings of inhibitor formed π−π stacking interactions with the imidazole ring of His41. It is once again confirmed that the cleavage of SARS-CoV-2 polyproteins by 3CLpro (3-Chymotrypsin-like protease) is facilitated by a Cys145–His41 catalytic dyad [105].

We hope that this review will be useful for the synthesis of other pyridine compounds with antimicrobial and antiviral properties, in order to help the current situation of the pandemic, with the infection with SARS-CoV-2.

## Figures and Tables

**Table 1:** In vitro antibacterial activity (minimal inhibitory/bactericidal concentrations, MIC and MBC), expressed in μM, for sulfonamides **36** and **37 [22]**.

Compound	Test	Gram-Positive			Gram-Negative		
		*B. subtilis*	*S.aureus*	*E. faecalis*	*E. coli*	*P. aeruginosa*	*Samonella typhi*
**36**	MIC	16.12	5.74	11.29	22.61	90.48	11.29
	MBC	30.63	10.71	19.19	36.16	171.89	19.19
**37**	MIC	10.11	20.26	40.52	20.26	72.05	48.02
	MBC	17.29	40.52	81.08	40.52	144.11	94.70
Tetracycline	MIC	70.31	14063	140.6	35.14	140.6	7.31
	MBC	91.40	196.8	210.9	42.16	196.89	98.44

**Table 2:** Antimicrobial activities of compounds **51**–**56** expressed as MIC values (mM) [31].

Compound	*A. niger*	*C. albicans*	*P. chrysogenum*	*S. aureus*	*B. subtilis*	*E. coli*	*P. aeruginosa*
**51**	-	12.497	-	6.2485	6.2485	6.2485	6.2485
**52**	12.499	3.125	12.4999	1.5625	1.5625	1.5625	1.5625
**53**	1.5625	0.7182	1.5625	0.1953	0.1953	0.3906	0.7812
**54**	0.3906	0.1953	0.1953	0.0488	0.0977	0.1953	0.1953
**55**	0.1953	0.0976	0.1953	0.0244	0.0244	0.0488	0.09764

**Table 3:** Minimum inhibitory concentrations (MIC, μg mL^−1^) and minimum bactericidal concentrations (MBC, μg mL^−1^) of the most active bis(indolyl)pyridines [38].

Compounds	Pathogenic Microorganisms					
	*S. aureus* ATCC 6538		*E. coli* ATCC 25922		*C. albicans* ATCC 10231	
	MIC	MBC	MIC	MBC	MIC	MBC
**77b**	78.125	312.5	312.5	1250	78.13	312.5
**78a**	39.063	625	312.5	625	39.06	156.25
**78c**	312.5	625	625	1250	39.06	78.13
**78d**	9.766	19.53	312.5	1250	19.53	19.53

**Table 4:** In vitro antimicrobial screening results of compounds **108a**–**108e**.

Compound	Minimum Inhibitory Concentration (MIC [μg mL^−1^])						
	Gram-Positive Bacteria		Gram-Negative Bacteria		Fungi		
	Sa	Sp	Ec	Pa	Ca	An	Ac
**108a**	100	62.5	62.5	50	250	100	100
**108b**	250	100	100	100	100	50	50
**108c**	100	50	50	50	250	100	100
**108d**	50	25	12.5	25	50	25	12.5
**108e**	25	50	50	25	100	50	50
Chloramphenicol	50	50	50	50	-	-	-
Ketoconazole	-	-	-	-	50	50	50

Sa: *Staphylococcus aureus* MTCC 96; Sp: *Staphylococcus pyogenes* MTCC 442; Ec: *Escherichia coli* MTCC 443; Pa: *Pseudomonas aeruginosa* MTCC 1688; Ca: *Candida albicans* MTCC 227; An: *Aspergillus niger* MTCC 282; Ac: *Aspergillus clavatus* MTCC 1323.

**Table 5:** Antimicrobial activity (MIC values in μg mL^−1^) of analogues **114a**–**114e**.

Compound	Gram-Positive Bacteria		Gram-Negative Bacteria		Fungi		
	Sa	Sp	Ec	Pa	Ca	An	Ac
**114a**	50	25	50	50	50	50	50
**114b**	25	25	25	25	50	25	25
**114c**	50	50	50	50	25	50	100
**114d**	25	50	25	25	50	25	50
**114e**	12.5	25	25	12.5	25	12.5	25
Chloramphenicol	50	50	50	50	-	-	-
Griseofulvin	-	-	-	-	500	100	100

Sa: *Staphylococcus aureus* MTCC 96; Sp: *Staphylococcus pyogenes* MTCC 442; Ec: *Escherichia coli* MTCC 443; Pa: *Pseudomonas aeruginosa* MTCC 1688; Ca: *Candida albicans* MTCC 227; An: *Aspergillus niger* MTCC 282; Ac: *Aspergillus clavatus* MTCC 1323.

**Table 6:** Antimicrobial activity (MIC values in μg mL^−1^) of pyridine triazoles **122a**−**122k [57]**.

Compd.	Bacterial Strains					Fungal Strains			
	Sa	Bs	Ec	St	Kp	An	Afu	Afl	Ca
**127a**	12.5	25	25	12.5	12.5	12.5	50	50	12.5
**127b**	12.5	12.5	25	25	6.25	12.5	6.25	25	6.25
**127c**	12.5	25	12.5	12.5	12.5	12.5	25	25	25
**127d**	6.25	12.5	6.25	12.5	25	25	25	25	25
**127e**	25	25	25	25	25	12.5	25	50	12.5
**127f**	25	25	12.5	25	12.5	12.5	25	25	12.5
**127g**	12.5	25	12.5	50	6.25	6.25	25	25	12.5
**127h**	12.5	12.5	6.25	25	6.25	6.25	25	12.5	12.5
**127i**	6.25	12.5	6.25	25	12.5	6.25	6.25	6.25	6.25
**127j**	6.25	12.5	6.25	25	12.5	6.25	12.5	6.25	6.25
**127k**	6.25	12.5	6.25	25	6.25	6.25	12.5	12.5	6.25
Ciprofloxacin	6.25	6.25	6.25	6.25	6.25	-	-	-	-
Miconazole	-	-	-	-	-	6.25	12.5	6.25	6.25

Sa: *Staphylococcus aureus* MTCC 096; Bs: *Bacillus subtilis* MTCC 441; Ec: *Escherichia coli* MTCC 443; St: *Salmonella typhi* MTCC 733; *Klebsiella pneumoniae* MTCC 432; An: *Aspergillus niger* MTCC 282; Afu: *Aspergillus fumigatus* MTCC 343; Afl: *Aspergillus flavus* MTCC 277; Ca: *Candida albicans* MTCC 227.

**Table 7:** Minimal inhibition concentration (μg mL^−1^) of hybrids **143a**–**143h** [61].

Compd.	Gram-Positive Bacteria				Gram-Negative Bacteria			Fungal Strain		
	Ec	Pa	Kp	St	Sa	Sp	Bs	Ca	An	Ac
**143a**	200	**125**	200	200	250	250	**100**	1000	500	500
**146b**	**100**	200	250	250	**200**	250	250	1000	500	500
**143c**	250	250	**100**	500	500	500	500	**200**	**200**	**250**
**143d**	150	150	200	200	**100**	**125**	**100**	500	>1000	>1000
**143e**	250	250	500	250	500	500	200	1000	>1000	>1000
**143f**	250	250	250	250	**200**	250	250	500	>1000	>1000
**143g**	500	500	**62.5**	500	**125**	200	250	**200**	**250**	**200**
**143h**	**50**	**100**	**100**	**100**	500	500	250	1000	1000	**62.5**
Ampicillin	100	100	100	100	250	100	-	-	-	-
Griseofulvin	-	-	-	-	-	-	-	500	100	100

Ec: *E. coli* (MTCC 443); Pa: *P. aeruginosa* (MTCC 1688); Kp: *Kl. pneumoniae* (MTCC109); St: *S. typhi* (MTCC98); Sa: *S. aureus* (MTCC 96); Sp: *S. pyogenus* (MTCC 442); Bs: *B. subtilis* (MTCC 441); Ca: *C. albicans* (MTCC 227); An: *A. niger* (MTCC 282); Ac: *A. clavatus* (MTCC 1323).

**Table 8:** Antimicrobial activities of the compounds **154** and **155** [66].

Compound	Inhibition Zone in mm (at 50 μg mL^−1^)			
	*B. subtilis*	*S. aureus*	*E. coli*	*C. albicans*
**154**	19	24	20	16
**155**	20	22	21	15
Ciprofloxacin	23	23	25	-
Ketoconazole	-	-	-	25

**Table 9:** Antimicrobial activities of the compounds **156**–**158** [67].

Compound	Inhibition Zone in mm (at 50 μg mL^−1^)				
	*B. subtilis*	*S. aureus*	*E. coli*	*C. albicans*	*A. niger*
**156**	1.55	1.60	0.80	0.65	1.60
**157**	1.35	1.75	0.60	0.75	1.90
**158a**	1.45	1.45	0.80	-	1.60
**158b**	0.90	1.30	-	0.65	1.70
**158c**	1.80	1.25	0.60	-	1.80
**158d**	0.85	1.30	0.75	0.65	1.95
**158e**	1.80	1.25	0.70	0.60	1.65
**158f**	1.60	1.45	0.70	0.55	.75
Ampicillin	1.15	1.30	0.75	-	-
Ketoconazole	-	-	-	0.80	2.30

**Table 10:** Minimum inhibitory concentration (MIC) in μg mL^−1^ of compounds **159**–**161**.

Compound	*S.aureus*	*B. subtilis*	*S. cerevisiae*	*C. albicans*
**159**	32	32	128	64
**160**	32	64	64	64
**161**	128	128	32	32
Ciprofloxacin	5	5	-	-
Amphotericin-B	-	-	20	20

**Table 11:** MIC values (in μg mL^−1^) of oxazolo[4,5-b]pyridine derivatives **169a**–**169h** [72].

Compound	Gram-Positive Bacteria		Gram-Negative Bacteria	
	*S. aureus* ATCC 2943	*B. subtilis* ATCC 6633	*E. coli* ATCC 13067	*P. diminuta* MTCC 3361
**169a**	6.25	25	50	25
**169b**	6.25	12.5	25	25
**169c**	3.125	6.25	25	12.5
**169d**	12.5	12.5	50	25
**169e**	6.25	25	50	50
**169f**	1.56	3.125	12.5	6.25
**169g**	3.125	6.25	12.5	12.5
**169h**	12.5	25	50	25
Ampicillin	6.25	12.5	50	25
Streptomycin	12.5	25	50	25

**Table 12:** MIC values of 4H-pyrimido[2,1-b]benzothiazoles **185a**–**185h** [79].

Compd.	Minimum Inhibitory Concentration (in μM mL^−1^)					
	*S. aureus*	*S. typhi*	*P. aeruginosa*	*E. coli*	*B. cereus*	*P. rettgeri*
**185a**	20	20	10	-	5	20
**185b**	20	-	20	-	5	-
**185c**	-	-	20	10	5	-
**185d**	-	20	10	5	2.5	10
**185e**	-	10	5	10	2.5	10
**185f**	1.25	20	10	5	2.5	-
**185g**	20	-	-	10	5	20
**185h**	-	-	20	20	5	10
Ciprofloxacin	1.25	2.5	1.25	1.25	0.62	1.25

**Table 13:** Minimum inhibitory concentrations (MICs) of α-aminophosphonates **200a**–**200e**.

Compound	Minimum Inhibitory Concentrations (MICs) (μg mL^−1^)				
	*E. coli*	*S. aureus*	*B. subtilis*	*C. albicans*	*S. cerevisiae*
**200a**	100	10	10	10	10
**200b**	10	10	10	10	10
**200c**	10	50	10	10	10
**200d**	10	10	10	10	10
**200e**	10	10	10	10	10
Ciprofloxacin	5	5	5	-	-
Amphotericin B	-	-	-	15	15

**Table 14:** In vitro antitubercular activity of compounds **220a** and **221** in comparison to some reference drugs.

Compound	MIC (μg mL^−1^)	Growth Inhibition, %
**220a**	6.25	75
**220b**	25	65
**220c**	3.12	80
**220d**	25	65
**221a**	12.5	70
**221b**	50	50
**221c**	50	60
**221d**	50	50
Izoniazid	0.6125	-
Pyrazinamide	3.125	-
Streptomycin	6.25	-
Ciprofloxacin	3.125	-

**Table 15:** In vitro inhibition of the growth on respective cell lines of adenovirus 5, herpesvirus 1, coxsackievirus B5, and echovirus 7 [102].

Compound	Antiviral Activity EC_50_ (μM)			
	HeLa		GMK	
	Adenovirus 5	Herpesvirus 1	Coxsackievirus B5	Echovirus 7
**230a**	>100	>100	1.7 ± 1.6	3.2 ± 3.3
**230b**	15.2 ± 31.4	>100	2.7 ± 4.6	033 ± 0.21
**230c**	>100	56.7 ± 61.3	4.3 ± 3.1	0.63 ± 0.38

## Data Availability

Not available.
